# Systematic comparison of small RNA library preparation protocols for next-generation sequencing

**DOI:** 10.1186/s12864-018-4491-6

**Published:** 2018-02-05

**Authors:** Cloelia Dard-Dascot, Delphine Naquin, Yves d’Aubenton-Carafa, Karine Alix, Claude Thermes, Erwin van Dijk

**Affiliations:** 1 Institute for Integrative Biology of the Cell, UMR9198, CNRS CEA Univ Paris-Sud, Université Paris-Saclay, 9198 Gif sur Yvette Cedex, France; 2 GQE – Le Moulon, INRA, Univ. Paris-Sud, CNRS, AgroParisTech, Université Paris-Saclay, 91190 Gif-sur-Yvette, France

**Keywords:** Small RNA, Bias, Library preparation, Next-generation sequencing, NGS, 2’-O-methyl RNA

## Abstract

**Background:**

Next-generation sequencing technologies have revolutionized the study of small RNAs (sRNAs) on a genome-wide scale. However, classical sRNA library preparation methods introduce serious bias, mainly during adapter ligation steps. Several types of sRNA including plant microRNAs (miRNA), piwi-interacting RNAs (piRNA) in insects, nematodes and mammals, and small interfering RNAs (siRNA) in insects and plants contain a 2’-O-methyl (2’-OMe) modification at their 3′ terminal nucleotide. This inhibits 3′ adapter ligation and makes library preparation particularly challenging. To reduce bias, the NEBNext kit (New England Biolabs) uses polyethylene glycol (PEG), the NEXTflex V2 kit (BIOO Scientific) uses both randomised adapters and PEG, and the novel SMARTer (Clontech) and CATS (Diagenode) kits avoid ligation altogether. Here we compared these methods with Illumina’s classical TruSeq protocol regarding the detection of normal and 2’ OMe RNAs. In addition, we modified the TruSeq and NEXTflex protocols to identify conditions that improve performance.

**Results:**

Among the five kits tested with their respective standard protocols, the SMARTer and CATS kits had the lowest levels of bias but also had a strong formation of side products, and as a result performed relatively poorly with biological samples; NEXTflex detected the largest numbers of different miRNAs. The use of a novel type of randomised adapters called MidRand-Like (MRL) adapters and PEG improved the detection of 2’ OMe RNAs both in the TruSeq as well as in the NEXTflex protocol.

**Conclusions:**

While it is commonly accepted that biases in sRNA library preparation protocols are mainly due to adapter ligation steps, the ligation-free protocols were not the best performing methods. Our modified versions of the TruSeq and NEXTflex protocols provide an improved tool for the study of 2’ OMe RNAs.

**Electronic supplementary material:**

The online version of this article (10.1186/s12864-018-4491-6) contains supplementary material, which is available to authorized users.

## Background

Small RNAs (sRNAs) are known to play an important regulatory role in a wide range of organisms in many biological processes including embryo development, cell differentiation, growth/proliferation and apoptosis/cell death [1]. Eukaryotic regulatory sRNAs typically range in size from ~ 20 to 30 nt and the three major classes are microRNAs (miRNA), small interfering RNAs (siRNA) and piwi-interacting RNAs (piRNA). Altered miRNA expression profiles have been implicated in a number of diseases [2], highlighting the importance of miRNAs in biology and the need for continued development of research tools for the study of sRNA in general.

Next-generation sequencing (NGS) is a powerful tool for the analysis of sRNAs. It has several advantages over microarray techniques or quantitative PCR (qPCR). It allows for the discovery of novel sRNAs, has a better signal to noise ratio than microarrays and does not suffer of saturation effects. In addition, it allows for the detection of single base differences and has a higher throughput than qPCR or Northern blotting. However, NGS approaches also have some disadvantages, such as the cost per sequencing run and the extensive processing steps required to convert a sample into a library for sequencing. In a typical sRNA library preparation process, adapters are ligated to the RNAs followed by reverse transcription and PCR amplification. All these steps are potential sources of bias [3, 4]. Consequently, read numbers may not reflect actual sRNA expression levels and different sRNAs may be either over- or underrepresented in the library. Strongly underrepresented sRNAs, especially when their actual expression levels are already low, may remain undetected. An additional problem arises with plant miRNAs, piRNAs in insects nematodes and mammals, and siRNAs in insects and plants in which the 3′ terminal nucleotide carries a 2’-O-methyl (2’ OMe) modification [1]. This modification strongly reduces the efficiency of 3′ adapter ligation [5], thus making library preparation particularly challenging for these types of sRNA.

Several studies demonstrated that adapter ligation steps may cause serious bias, due to RNA sequence/structure effects resulting in the preferential ligation of certain sRNAs with a given adapter sequence, while others are disfavoured [6–11]. We will refer to this type of bias as “sequence bias”. Randomisation of adapter sequences close to the ligation junction would neutralize this effect and improve the fidelity of NGS results. Sorefan and colleagues [7] used adapters with 4 random nucleotides at the ligation junctions and named these “High Definition” (HD) adapters. A more recent study revealed that the randomised region does not have to be adjacent to the ligation junction and in addition showed that the use of a 5′ adapter that has a region complementary to the 3′ adapter can promote the formation of structures favourable for ligation [11]. This novel type of adapters was designated “MidRand” adapters. Together, these studies demonstrate that bias can be reduced through improved adapter design.

Instead of modifying the adapters, some other studies sought to suppress bias through the optimisation of reaction conditions. Several laboratories tested a thermostable DNA/RNA ligase, ‘MthRnl’ from New England Biolabs that works at 65 °C, a temperature at which short RNAs should be largely unstructured. Even though one study reported bias reduction by this enzyme [12], two others did not observe any effect [13, 14]. Moreover, Song and collaborators tried ligation at 18 °C, 25 °C, or 37 °C with a truncated version of the classical RNA ligase 2, and best results were obtained at 25 °C [14]. In addition, these authors and Zhang et al. [12] found that polyethylene glycol (PEG), a macromolecular crowding agent known to increase ligation efficiency [15], led to a significant reduction of bias. Song and collaborators found an optimal capture efficiency at 15–25% PEG but, surprisingly, the optimal PEG concentration was not the same for all miRNAs tested [14]. Based on these results, New England Biolabs (NEB) released the “NEBNext” kit, which uses PEG in the ligation reactions (in combination with classical Illumina adapters) and BIOO Scientific has released the “NEXTflex sRNA library preparation kit” that combines the use of HD adapters with PEG and is advertised as a kit with less bias than the traditional kits.

However, all these results were obtained with unmodified RNAs and do not necessarily extrapolate to 2’ OMe RNAs. One paper reported that the addition of 25% PEG restored ligation efficiency of a 2’-OMe modified sRNA to the level of the same RNA without modification [5]. However, it remains to be determined if PEG will restore the ligation efficiency of 2’-OMe RNA in a complex mixture where competition effects will occur. In addition, the effects of different adapters with- or without randomized regions on the representation of 2’-OMe RNAs in NGS libraries have not been examined.

Another concern for sRNA library preparation is the formation of side products such as adapter dimers. Currently available library reparation kits either use strategies to eliminate excess 3′ adapter before 5′ adapter ligation, including purification steps or the use of complementary oligonucleotides that inactivate the 3′ adapter. Several alternative approaches have been developed. For example, Shore et al. [16] developed a method in which the 5′ and 3′ adapters carry chemical modifications that block dimer formation, while allowing for efficient ligation of the adapters with small RNAs. Alternative approaches use blocking oligonucleotides that are complementary to the adapter dimer ligation products [17] or the single stranded DNA-specific exonuclease RecJ to degrade non-ligated 3′ adapters [18].

Some protocols use polyadenylation instead of ligation for 3′ adapter addition. Multiple A residues are added to the 3′ end using poly(A) polymerase (PAP) [3]. Then, a 5′ adapter is ligated either directly to the RNA or to the nascent cDNA after reverse transcription. Novel sRNA library preparation kits from Clontech (SMARTer smRNA-seq) and Diagenode (CATS Small RNA-seq kit) even avoid adapter ligation altogether, as a 3′ adapter is added by polyadenylation and a 5′ adapter is added through reverse transcriptase template-switching. Thus, ligation bias is avoided. However, PAP can also be affected by RNA structures and 2’ OMe modifications can reduce the efficiency of polyA tailing [5]. It remains therefore to be seen if this method will perform better than the classical ligation-based approaches.

In this study, we systematically compared the classical TruSeq kit from Illumina with four commercially available ‘low bias’ kits, the NEBNext kit from NEB, the NEXTflex V2 kit from BIOO Scientific, the SMARTer kit from Clontech and the CATS kit from Diagenode. We tested the performance of these kits with regard to three parameters: (1) bias among sequences, (2) bias against 2’ OMe RNAs, and (3) the formation of side products. The ideal protocol should have low overall bias and generate few side products. This would allow to faithfully reproduce true sRNA expression profiles and to capture (weakly expressed) sRNAs that might otherwise escape detection. We tested the kits with and without several modifications, and using human miRNAs as representatives of non-modified sRNAs and plant miRNAs representing 2’ OMe-modified sRNAs. Our results identify protocols that work best for these different types of sRNA. Our modifications lead to better detection of 2’ OMe RNAs than the standard protocols.

## Results and discussion

### Experimental strategy

**Fig. 1:**
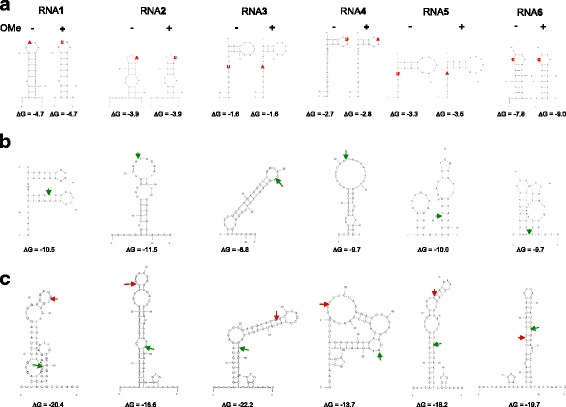
**a** Predicted secondary structures of the synthetic sRNAs 1–6 used in this study (Methods). The free energies at 28 °C are indicated for each sRNA without or with 2’ OMe. Nucleotide substitutions introduced to distinguish between the unmodified RNAs (RNA1–6) and the 2’OMe variants (RNA-OMe1–6) are indicated in red. These nucleotide substitutions did not alter the predicted secondary structures. The absence or presence of 2’ OMe modification is indicated by “-”or “+” signs, respectively. **b** Predicted secondary structures of RNAs1–6 ligated with the Illumina 3′ adapter. The ligation junctions are indicated by green arrows. **c** Predicted secondary structures of RNAs1–6 ligated with both the Illumina 3′ and 5′ adapter. The 3′ ligation junctions are indicated by green arrows, the 5′ ligation junctions are indicated by red arrows. Note that for 3′ adapter ligation the structures in (**b**) should be considered and for subsequent 5′ adapter ligation the structures in (**c**)

To test the performance of the various kits, we designed a pool of six synthetic sRNAs, supplemented with random 21 nt RNAs to create a complex mixture. As ligation efficiency can be influenced by RNA structure effects, the six synthetic sRNAs (RNAs1–6) were designed with various predicted secondary structures (Methods, Fig. 1). We thus generated a pool with representatives of different types of sRNA that would enable us to examine a possible relation between levels of secondary structure formation and representation in the library. Of each of these RNAs a 2’-OMe variant was generated (RNA-OMe1–6). To distinguish between the 2’-OH and the 2’OMe variants, we introduced a single nucleotide substitution that did not affect the predicted secondary structure. This was important to make sure because differences in representation between 2’-OH and the 2’OMe variants might otherwise be due to RNA structure bias rather than to bias against the 2’OMe variants. To create a complex mixture, RNAs(OMe)1–6 were added to a mixture of synthetic random sRNAs to a final molar concentration of 1% for each RNA. Libraries were constructed using the TruSeq (TS), NEXTflex (Nf), NEBNext (NN), SMARTer (S), or CATS (C) kit. In the TS and Nf protocols, we introduced a series of modifications as detailed in Table 1. The resulting libraries were sequenced using Illumina technology. We determined the proportion of sequences corresponding to RNAs(OMe)1–6 for each protocol and we tested their performance regarding the following three parameters: (1) sequence bias, (2) 2’-OMe bias, and (3) formation of side products.

**Table 1:** Overview of the protocols used in this study

kit used	protocol	modifications
Illumina TruSeq	TS1	* none: standard conditions*
	TS2	HD adapters used instead of Illumina adapters
	TS3	3′ adapter ligation 16 °C o/n in the presence of PEG
	TS4	HD adapters used instead of Illumina adapters
		3′ adapter ligation 16 °C o/n in the presence of PEG
	TS5	HD adapters used instead of Illumina adapters
		3′ adapter ligation 16 °C o/n in the presence of PEG
		purification step after 3′ adapter ligation
	TS6	MRL adapters used instead of Illumina adapters
	TS7	MRL adapters used instead of Illumina adapters
		3′ adapter ligation 16 °C o/n in the presence of PEG
		purification step after 3′ adapter ligation
BIOO Scientific NEXTflex V2	Nf1	* none: standard conditions*
	Nf2	3′ adapter ligation 16 °C o/n
	Nf3	Illumina adapters instead of BIOO Scientific (HD) adapters
		3′ adapter ligation 16 °C o/n
	Nf4	no PEG in the ligation reactions
	Nf5	MRL adapters used instead of BIOO Scientific adapters
		no PEG in the ligation reactions
	Nf6	MRL adapters used instead of BIOO Scientific adapters
		3′ adapter ligation 16 °C o/n
New England Biolabs NEBNext	NN	* none: standard conditions*
Clontech SMARTer	S	* none: standard conditions*
Diagenode CATS kit	C	* none: standard conditions*

The Illumina TruSeq (‘TS’), BIOO Scientific NEXTflex V2 (‘Nf’) or Clontech SMARTer (‘S’) kits were used following their respective standard protocols or variants thereof. The different variants of the protocols are distinguished by numbers and their respective modifications are indicated

### Comparison of biases introduced by the following kits: TruSeq, NEXTflex V2, NEBNext, SMARTer and CATS

**Fig. 2:**
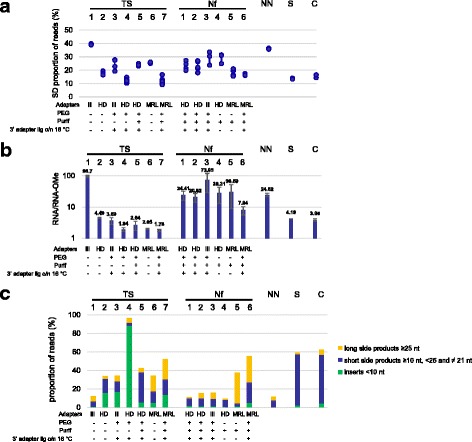
**a** Sequence bias of the various protocols. The standard deviation of the proportion of reads corresponding to each of the unmodified RNAs 1–6 was taken as a measure of sequence bias. Shown are the data for each replica of the different protocols. We did not consider variation among the 2’ OMe RNAs here, as additional variability is introduced by 2’ OMe bias. The type of adapters used for the various protocols and the presence (“+”) or absence (“-”) of PEG and a purification step after 3’ adapter ligation is indicated. Also the presence or absence of overnight ligation at 16 °C is indicated, with “absence (-)” meaning standard ligation at 28 °C (TS protocols) or 22 °C (Nf protocols). **b** 2’ OMe bias of the various protocols. The ratios of the total numbers of reads for the unmodified RNAs (RNA1–6) and for the 2’ OMe RNAs (RNA-OMe1–6) were determined for each protocol and in each separate experiment. Shown are the mean values of at least two independent experiments and the standard deviations are indicated by error bars. **c** Percentage of the total numbers of reads corresponding to side products. The percentages of raw reads with inserts < 10 nt (considered adapter dimers and eliminated after trimming) are indicated in green. Blue bars represent inserts ≥10 nt, < 25 and ≠ 21 nt that did not correspond to RNA(OMe)1–6. Yellow bars represent inserts ≥25 nt that did not correspond to RNA(OMe)1–6. Shown are the mean values of at least two independent experiments and the standard deviations are indicated by error bars

We started by comparing the levels of bias introduced by the various kits following their respective standard protocols. We determined the relative proportion of reads corresponding to each of the RNAs(OMe)1–6 considering the total numbers of raw reads before adapter trimming to take into account also potential losses of sequences due to the formation of adapter dimers (Additional file 1: Figure S1). To measure specifically the variability among sequences, i.e. sequence bias, we took the following approach: here we considered only the reads mapping to RNAs1–6 and we determined the proportion of these reads corresponding to each RNA. The results are presented in a box plot (Additional file 2: Figure S2); in addition we used the standard deviations of the read distributions obtained with the different kits as an easier to read measure of variability (Fig. 2a). To measure only sequence bias at this stage and not bias due to the 2’ OMe modification, we considered only the unmodified RNAs for this analysis.

The TruSeq kit used under standard conditions presented strong sequence bias (Fig. 2a and Additional file 1: Figure S1, protocol TS1), consistent with previous observations [6, 11, 12]. While RNA1 was ~ 10-fold overrepresented, RNAs 2, 3 and 5 were about 10-fold under-represented and RNAs 4 and 6 were even more than 100-fold underrepresented (Additional file 1: Figure S1). We checked if these observations were consistent with RNA secondary structure formation, creating either a favourable or an unfavourable context for ligation. It should be mentioned here that data from Sorefan and collaborators suggested that RNA ligase 2 (Rnl2), the enzyme used for 3′ adapter ligation, has a preference for a double-stranded environment directly upstream of the ligation site. In contrast, RNA ligase 1 (Rnl1), the enzyme used for 5′ adapter ligation, would prefer a single stranded context [7]. The predicted secondary structures (Fig. 1) are in partial agreement with this hypothesis and with our observations. For example, RNA1 is predicted to have a double stranded context at the 3′ adapter ligation site and a single stranded environment at the 5′ site, which is consistent with the observation that RNA1 is overrepresented. The other RNAs are predicted to have unfavourable structures for either 3′ or 5′ adapter ligation, consistent with these RNAs being under-represented. For the most under-represented RNAs 4 and 6, these structures predict a single stranded environment around both the 3′ and the 5′ ligation sites for RNA4, while for RNA6 double stranded structures are predicted at both sites. This would suggest that RNAs 4 and 6 would have poor 3′ or 5′ adapter ligation, respectively. To test this hypothesis, we ran a denaturing acrylamide gel with 3′ and 5′ adapter ligation products for RNAs 4 and 6 (Additional file 3: Figure S3). For comparison, we did the same analysis for RNA1, where both ligation steps should be efficient, and for RNA1-OMe, where 3′ adapter ligation is expected to be inhibited. For RNA4 we expected a similar phenotype as for RNA1-OMe, while for RNA6 we expected the opposite. The results shown in Additional file 3: Figure S3 are in agreement with our expectations for all RNAs except for RNA4. Here, like for RNA6, 5′ adapter ligation seems inhibited rather than 3′ ligation.

Thus, while it remains difficult to predict with precision the efficiency of 3′ and 5′ adapter ligation, it may be possible to estimate roughly whether a given RNA of interest will be well represented or not using the classical Illumina protocol. These results also illustrate that different RNAs can have similar levels of under-representation but for different reasons, i.e. due to either poor 3′ or 5′ adapter ligation efficiency. Thus, the observation by Song and colleagues [14] that optimal PEG concentrations differ among miRNAs may be explained by the fact that in some cases 3′ ligation, and in other cases 5′ ligation needs optimisation, with optimal PEG concentrations that may be different for both ligation reactions. Indeed, Zhang and colleagues used 10% PEG for 3′ adapter ligation and 20% PEG for the 5′ ligation reaction [12].

Next, to measure 2’ OMe bias, we determined the ratio of the mean values obtained for the collection of unmodified RNAs to the mean values obtained for the 2’ OMe RNAs. Protocol TS1 had a strong overall 2’ OMe bias, with an almost 100-fold average lower representation of the 2’ OMe RNAs than the unmodified RNAs (Fig. 2b). Strikingly, this bias varied dramatically from one RNA to another, ranging from about 3-fold (RNA5) to more than 300 fold (RNA3) (Additional file 1: Figure S1). There was no apparent correlation between the levels of sequence bias and 2’ OMe bias and it thus remains unclear what may cause such variability. Together, the combination of the two types of bias result in a more than 100,000-fold difference in capture efficiency between the most strongly detected RNA (RNA1) and the most weakly detected RNA (RNA6-OMe).

Less bias was observed with the NEXTflex protocol. However, there was still significant variation in detection efficiency among RNAs 1–6 (Fig. 2a and Additional file 1: Figure S1, Nf1) and a 20-fold less efficient detection of the 2’ OMe RNAs (Fig. 2b, Nf1). For a better detection of 2’ OMe RNAs the manufacturer recommends performing the 3′ adapter ligation overnight at 16 °C instead of 2 h at 22 °C (standard conditions). However, very similar profiles were obtained (Additional file 1: Figure S1, compare Nf1 with Nf2) and no difference in sequence- or 2’ OMe bias was observed (Fig. 2a and b, compare Nf1 with Nf2). It is thus at the user’s convenience to perform 3′ adapter ligation either 2 h at 22 °C or overnight at 16 °C. For practical reasons, we performed all 3′ adapter ligations with PEG overnight at 16 °C (except for Nf4, where PEG was omitted); we will thus consider protocol Nf2 as the standard NEXTflex protocol in the subsequent sections.

The NEBNext kit had somewhat less sequence bias than TruSeq, likely owing to the presence of PEG in the ligation reactions, but more than NEXTflex (Fig. 2a, compare protocols TS1 and NN). The 2’ OMe bias was similar to that observed with the NEXTflex protocol (Fig. 2b, compare protocols Nf1 and NN).

The SMARTer and CATS protocols performed very similarly and had both less sequence- and 2’ OMe bias than NEXTflex. The remaining sequence bias (Fig. 2a, protocols S and C) can be explained by the observation that, as for adapter ligation, RNA secondary structure influences the efficiency of 3′ end tailing [19]. In addition, the 2’ OMe modification can reduce the efficiency of polyadenylation by PAP [5]. Thus, even though the SMARTer and CATS protocols do not rely on ligation for adapter addition, a certain level of bias remains. An additional problem with these protocols was the formation of side products; while the formation of adapter dimers (inserts < 10 nt) was modest, as with the TruSeq and NEXTflex protocols, there was an abundant formation of short inserts (10–20 nt and 22–24 nt) that did not correspond to RNA(OMe1–6) or to the 21 nt random RNAs (Fig. 2c, protocols S and C). We examined the size distribution of these products and if there were sequences that predominated. There was a peak at 20 nt but no prevalent sequences were observed (data not shown). This may be explained by the fact that a 3′ A-tail is added followed by reverse transcription from an anchored oligo dT primer. Random RNAs that terminated by one or multiple A-residues will be reverse transcribed from the last non-A residue, resulting in a shorter product. Thus one fourth of the short side products may originate from random RNAs terminating by one A, and one sixteenth from RNAs terminating by two A’s. The remaining ~ 20% may be explained, at least in part, by incomplete synthesis products. It should be mentioned that RNA(OMe)2 and 4 also end by an A and would escape detection if, after trimming, a perfect mapping to the entire length of the RNA sequence were required. These RNAs were therefore mapped to the truncated sequence without the 3′ terminal A.

It is important to note that there are strong differences in the expression profiles produced by the different methods (Additional file 1: Figure S1, compare TS1, Nf1, NN, S, and C). These results are in agreement with previous observations by Baran-Gale and colleagues [20], and underscore the existence of method-specific sequence biases. It is likely that the use of different adapter sequences plays a role in these profile changes, as previously suggested [14]. However, our results presented below indicate that other factors can cause such changes as well. Thus, although the NEXTflex, NEBNext, SMARTer and CATS kits give a more faithful picture of the true expression levels of sRNAs, even these methods are still biased. As a result, it remains difficult to quantitatively compare the expression levels of different sRNAs.

### Evaluation of RT and PCR as potential sources of bias

Adapter ligation or polyadenylation is followed by reverse transcription and PCR amplification, two potential additional sources of bias. In two previous studies, steps downstream of adapter ligation did not play a significant role in bias among sequences [6, 11]. However, it is also known that PCR polymerases differ significantly among each other in terms of bias introduction and it thus remains to be established whether these results can be generalised to other sRNA library preparation protocols that rely on different polymerases. The Phusion polymerase provided with the Illumina kits is particularly bias-prone [21]. Instead of this enzyme, we therefore used the Kapa HiFi polymerase, which introduces much less bias [21], for the TruSeq libraries. For the NEXTflex and SMARTer libraries however, the enzymes provided with the kit (Duro Taq and AmpliTaq respectively) were used. To our knowledge, potential bias due to these enzymes had not been tested before. We thus amplified a standard TruSeq, NEXTflex and a SMARTer library for a total of 50 additional cycles with sample dilution every 10 cycles using Kapa HiFi polymerase, DuroTaq or AmpliTaq, respectively. No profile changes were observed even after 50 additional cycles of PCR with all three polymerases, arguing against a potential role for PCR in the bias observed with these libraries (Additional file 4: Figure S4).

While PCR does not appear to introduce bias in sRNA libraries, reverse transcription was reported to be inhibited by the 2’ OMe modification; in fact, this feature has been used to map methylation sites by truncation of reverse transcriptase extension products [22]. Using AMV- or excess amounts (up to 200 units) of M-MuLV reverse transcriptase reduced this effect [5]. The TruSeq, NEXTflex and SMARTer protocols all use at least 200 units of (variants of) the M-MuLV enzyme, and therefore reverse transcriptase effects are expected to be limited. We nevertheless tested if, when using AMV instead of M-MuLV reverse transcriptase the representation of 2’ OMe RNAs could be improved. A library was prepared following the classical TruSeq protocol but using AMV reverse transcriptase. No significant profile change was observed and bias against 2’ OMe RNAs did not decrease (Additional file 4: Figure S4).

### Evaluation of the individual contribution of HD adapters and PEG to bias reduction

As shown above, the NEXTflex protocol has less bias than the TruSeq protocol. While this is expected to be due mainly to the use of HD adapters and PEG for the ligation reactions, their relative contribution has not been evaluated. In addition, it remains possible that other factors play a role in the difference between the two kits. To address these questions, we modified both protocols as follows. In the TruSeq protocol, the classical Illumina adapters were replaced by HD adapters while leaving the other parameters unchanged (TS2), PEG was added to the ligation reactions to a final concentration of 20% in combination with the Illumina adapters (TS3), or in combination of HD adapters (TS4). With the NEXTflex protocol, the reverse experiment was performed; the HD adapters were replaced by Illumina adapters (Nf3) or PEG was left out of the reaction mixtures while keeping the HD adapters (Nf4). Note here that the standard NEXTflex protocol uses HD adapters in combination with PEG.

For both kits, HD adapters present less sequence bias than Illumina adapters, as previously reported [7–11] (Fig. 2a, compare protocols TS1 with TS2 and Nf2 with Nf3). Somewhat surprisingly, they also reduce overall 2’ OMe bias (Fig. 2b compare protocols TS1 with TS2 and Nf2 with Nf3). As shown in Additional file 1: Figure S1, for each RNA (except for RNA5 in TS2) the difference in representation of the unmodified- and 2’ OMe variant was less with HD adapters than with standard Illumina adapters (compare protocols TS1 with TS2 and Nf2 with Nf3). A likely hypothesis to explain reduced sequence bias due to HD adapters is that they would neutralize preferential ligation of certain RNAs with a given adapter due to favourable co-folds, while RNAs with unfavourable structures would be ligated less efficiently. It is difficult to apply the same model to explain reduced 2’ OMe bias since the 2’-OMe modification is not expected to affect RNA structure. Nonetheless, while the exact mechanism remains unclear, HD adapters substantially improve the detection of 2’-OMe RNAs.

PEG reduced, but did not eliminate 2’ OMe bias and sequence bias with both kits (Fig. 2a, b and Additional file 1: Figure S1, compare TS1 with TS3, and Nf2 with Nf4). Our results thus showed a smaller effect of PEG on bias against 2’-OMe RNAs than in the study by Munafo and Robb [5], where only a single sRNA sequence was tested. In this light, it is important to note that bias against 2’-OMe RNAs strongly depends on the RNA sequence; while for RNA4 and 5 there was an almost equal detection of the unmodified and the 2’OMe variants (Additional file 1: Figure S1, protocol TS3), substantial bias remained for the other RNAs. Thus, by using a pool of different RNAs, our study provides a more complete picture of the effect of PEG on bias against 2’-OMe RNAs, and in addition confirms that PEG reduces sequence bias.

However, as compared with the standard TruSeq protocol, the use of HD adapters or the addition of PEG also led to the formation of numerous side products, mainly consisting of adapter dimers or short inserts (10–20 nt and 22–24 nt) that did not correspond to any of the RNA(OMe1–6) or to the 21 nt random RNAs (Fig. 2c, TS2 and − 3).

The combined use of HD adapters and PEG following the Illumina protocol further reduced bias (Fig. 2a and b, TS4). However, there was an excessive formation of adapter dimers (Fig. 2c, TS4). It was striking to see that the combination of HD adapters and PEG led to a strong accumulation of adapter dimers with the TruSeq kit, while the NEXTflex kit produced very small amounts of adapter dimers (Fig. 2c, compare TS4 with Nf2). It should be mentioned here that different strategies are used by the two protocols to reduce the formation of adapter dimers. The TruSeq protocol uses a ‘STOP’ oligonucleotide that hybridises to the 3′ adapter to prevent ligation of unligated 3′ adapter with the 5′ adapter. The NEXTFlex protocol instead includes a purification step after 3′ adapter ligation to remove excess 3′ adapter. Adding this step to the TruSeq protocol efficiently reduced the levels of adapter dimers, but the formation of other short side products increased (Fig. 2c, compare TS4 with TS5). Alternative solutions to reduce adapter dimers also exist. For example, Xu and colleagues, who also observed an abundant formation of adapter dimers in the presence of PEG and HD adapters, published a protocol that uses the exonuclease RecJ to eliminate excess 3′ adapter before 5′ ligation [18].

Intriguingly, the addition of a purification step led to increased bias as compared to the protocol without this step (Fig. 2a, b, compare TS4 with TS5). In addition, there was a marked profile change (Additional file 1: Figure S1, compare TS4 with TS5). Importantly, this result illustrates that even when using the same adapters, the addition of a simple purification step can substantially affect expression profiles. This highlights the importance to use exactly the same protocol for a series of samples that are to be compared.

Both sequence bias as well as 2’ OMe bias remained lower than with the NEXTflex protocol (Fig. 2a and b, compare TS5 with Nf2), raising the question of what may cause this difference. In both protocols a very similar procedure is followed for 3′ adapter ligation (see Methods for details), but the concentration of PEG is 20% in the TruSeq protocol, against 12% in the NEXTflex protocol. This is likely to contribute to the lower 2’ OMe bias observed with the TruSeq protocol, since it has been shown previously that the use of 25% PEG better reduces 2’ OMe bias than the use of 12.5% PEG [5].

In conclusion, HD adapters and PEG have roughly similar effects, i.e. they both reduce sequence bias as well as 2’ OMe bias. The combination of HD adapters and PEG with a purification step had less bias with the TruSeq kit (TS5) than with the NEXTflex kit (Nf2). Our results further indicate that small changes in protocols, even when keeping the same adapters, can have profound effects on expression profiles.

### Comparison of bias with HD adapters and MRL adapters

Fuchs and colleagues developed a variant of HD adapters, called ‘MidRand’, in which the randomised regions are in the middle of the adapters rather than at the extremities [11]. In addition, there is a region of complementarity between the 3′ and 5′ adapter, which may help to improve ligation efficiency. These authors reported that MidRand adapters reduce sequence bias, indicating that the randomised region does not have to be close to the ligation junction. However, whether this type of adapters performs better or not than HD adapters has not been addressed. Here we use a variant of MidRand adapters, which we call MidRand-Like (MRL) adapters, with the following changes: (1) the sequences of both adapters were adjusted to make them compatible with the TruSeq/NEXTflex reverse transcription- and PCR primers, so that they can easily be used with these kits. In addition, (2) the 5′ adapter was a chimera of DNA and RNA instead of all RNA to make it less sensitive to degradation and less prone to form secondary structures. We used MRL adapters both in the absence (TS6 and Nf5) as well as in the presence of PEG (TS7 and Nf6). Our data indicate that MRL adapters reduce bias as compared with Illumina adapters (Fig. 2a and b, compare TS6 with TS1). However, as compared with HD adapters, no clear further reduction of bias is observed with MRL adapters in the absence of PEG (Fig. 2a and b, compare TS6 with TS2 and Nf5 with Nf4). When MRL adapters are combined with PEG, a more consistent bias reduction is observed as compared with HD adapters and PEG (Fig. 2a and b, compare TS5 with TS7 and Nf2 with Nf6). While using the TruSeq kit the percentage of side products did not change significantly, with the NEXTflex kit the formation of side products increased from 10 to 15% with HD adapters to about 50% with MRL adapters (Fig. 2c, compare Nf2 with Nf6). A substantial proportion of these side products were “long side products” (larger than 25 nt after trimming) and contained partial 3′ adapter sequences, either or not preceded by a sequence corresponding to RNA(OMe)1–6 or an unidentified sequence, for which trimming was unsuccessful. All adapters used in this study were checked on polyacrylamide gels (see Methods), and no faster migrating species were seen for the MRL adapters. It is therefore at present not clear what these partial adapter sequences originate from.

### Bias does not prevent the quantitative detection of sRNAs with or without 2’ OMe

The strong bias observed with the classical TruSeq protocol raises questions of the capacity to detect expression changes of a given sRNA in different conditions. It was previously reported that despite strong sequence bias 10–1000 fold changes were well detected [6], and a similar fold change among conditions was detected using either Illumina or HD adapters [7]. Thus, sequence bias does not appear to prevent the identification of differentially expressed sRNAs. However, these studies examined relatively large fold changes and a potential influence of 2’ OMe bias has not been investigated. We tested the capacity of protocols TS1, TS5, Nf2, and S to detect small (2–10)-fold changes in abundance of RNAs 1–6 and RNA-OMe1–6 (Additional file 5: Figure S5). We prepared libraries from the synthetic RNA mixture described above, with RNAs1–6 and RNA-OMe1–6 at 1% molar concentration each (mix A), or from a mixture with altered levels of each RNA (mix B). All four protocols generally detected the theoretical fold changes for most RNAs quite well, irrespective of the absence or presence of the 2’ OMe modification, with coefficients of determination ranging from 0.83 to 1.00. Even the 2- and 0.5-fold expression changes of RNA(OMe)4 and 5 were accurately detected by TS1, indicating that the strong bias with this protocol does not lead to a less quantitative detection of sRNAs with or without 2’ OMe. However, for some RNAs detection may be more accurate than for others. Finally, it should be noted that even though 10 million reads per library were generated in this experiment, only a few reads were obtained for the most weakly expressed RNAs with the TruSeq protocol, while for the other protocols at least 80–100 reads corresponded to the most weakly detected RNA. It therefore follows that, especially in the case of 2’ OMe RNAs, the TruSeq protocol will require sequencing at much greater depth than the other protocols for accurate quantitative detection.

To further confirm these observations, we selected a few protocols (TS7, Nf2, and Nf6) to prepare libraries using *B. napus* sRNA preparations from either stems and leaves or from flower buds, and miRNA expression changes were measured. As shown in Additional file 6: Figure S6, similar fold changes were obtained using the different protocols.

To summarise the above sections, our analysis with the synthetic RNAs revealed that the SMARTer and CATS kits had less bias than the standard TruSeq and NEXTflex protocols. However, upon modification, bias levels with the TruSeq kit strongly decreased and dropped below those obtained with the SMARTer and CATS kits. MRL adapters introduce less bias than HD adapters with both the TruSeq and NEXTflex kits. Unfortunately, low bias conditions also favoured the formation of side products. There was a striking reverse correlation between bias levels and side product formation, and the performance of a protocol will thus depend on a balance between these two. For 2’ OMe RNAs there is, next to sequence bias, also 2’ OMe bias to be taken into account in this balance. As a result, protocols that perform best for unmodified RNAs may not be the same as those that yield the best results for 2’ OMe RNAs.

### Comparison of miRNA detection in biological samples using the different protocols

**Fig. 3:**
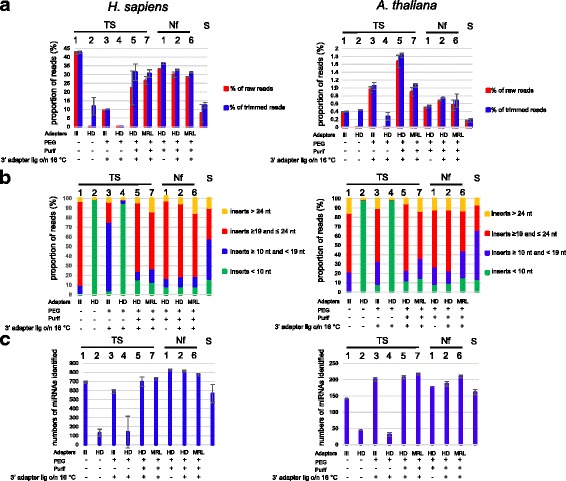
**a** Percentage of reads mapping to human or *Arabidopsis* miRNAs. The proportion of reads mapping to human miRNAs (unmodified) or *Arabidopsis* miRNAs (with 2’ OMe modification) in miRBase were determined for the different TruSeq protocols (TS1–6), the NEXTflex protocols (Nf1–5), and the SMARTer protocol (S). We calculated the percentage of the total numbers of raw reads (red bars) or the total numbers of reads after trimming (blue bars) that mapped to miRNAs. Shown are the mean values of at least two independent experiments and the error bars represent standard deviations. The histograms on the left and on the right show the results for human or *Arabidopsis* libraries, respectively. The type of adapters used for the various protocols and the presence (“+”) or absence (“-”) of PEG and a purification step after 3′ adapter ligation is indicated. **b** Percentage of informative reads and side products. Following adapter trimming, the obtained reads were subdivided in four size categories: (1) inserts < 10 nt, indicated by green bars, (2) inserts ≥10 nt and < 19 nt, (3) inserts ≥19 and ≤24 nt, and (4) inserts > 24 nt. Given de size distribution of miRNAs, the third category was considered to contain informative reads, while the others may contain side products. Shown are the mean values of at least two independent experiments. The histograms on the left and on the right show the results for human or *Arabidopsis* libraries, respectively. **c** Numbers of known human or *Arabidopsis* miRNAs identified. We determined the numbers of known miRs identified with the various protocols. For each protocol, one million of reads were trimmed and the 19–24 nt inserts were used for mapping to human or *Arabidopsis* miRNAs in miRbase. Shown are the mean values of at least two independent experiments with standard deviations represented by error bars. The histograms on the left and on the right show the results for human or *Arabidopsis* libraries, respectively

We next wished to determine how the various protocols would perform with biological samples. Our experimental strategy was to compare these protocols for the detection of either human miRNAs, that are unmodified, or *Arabidopsis* miRNAs, that are known to contain a 2’OMe modification. We assessed the protocols for three different aspects: (1) the percentage of sequencing reads corresponding to miRNAs, (2) the formation of side products, and (3) the number of different (i.e. unique) miRNAs detected. The libraries were sequenced using Illumina technology and the reads were trimmed for adapter sequences and mapped to human or *Arabidopsis* miRNA databases (miRBase; see Methods for details). Adapter trimming removed all sequences with inserts shorter than 10 nt. We considered inserts from 19 to 24 nt as potential miRNAs and used this size range for mapping, while inserts of 10 nt to 19 nt or longer than 24 nt were considered side products (technical noise or biological material other than miRNAs). Mapping was done with Bowtie [23], considering only perfect matches. The percentage of the total numbers of reads mapping to miRNAs for the various protocols is shown in Fig. 3a. With the standard TruSeq protocol (TS1), ~ 42% of the reads mapped to human miRNAs. While we did not expect an increased percentage of mapping with the reduced bias protocols, a decrease was observed. This can be explained largely by a loss of sequences due to an increased formation of adapter dimers and other side products (Fig. 3b). Especially in the case of protocols TS2 and TS4, there was an excessive formation of adapter dimers, leading to an almost complete loss of informative sequences. For protocols TS3 and S there was also a substantial loss of informative sequences, but mainly due to the formation of short side products. In the case of protocol TS3 these consisted mainly of fragments of the Illumina STOP oligonucleotide, which is added after 3′ adapter ligation. In the case of protocol S these products were mainly biological RNAs other than miRNAs, potentially degradation intermediates of larger RNA species (Additional file 7: Figure S7).

Strikingly, for *Arabidopsis* only ~ 0.4% of reads mapped to miRNAs with protocol TS1. In contrast to the human miRNAs, the percentage of reads corresponding to *Arabidopsis* miRNAs increased with the other protocols, likely owing to reduced 2’ OMe bias, except for protocols TS2, TS4 and S. Here, again there was a massive loss of sequences due to adapter dimers (TS2, TS4) and short side products (S). With all protocols, the percentage of mapped reads remained modest, which is consistent with literature data [18, 24]. This probably reflects, at least in part, remaining bias against 2’-OMe RNAs. Also with the synthetic RNAs, the 2’ OMe variants remained less represented than the unmodified RNAs even with the best performing protocols, but the difference was less pronounced (Fig. 2b and Additional file 1: Figure S1). Thus, it is possible that in the biological samples used here remaining bias against 2’ OMe RNAs is stronger than in the synthetic mix, or the actual amounts of miRNAs may be lower in the *Arabidopsis* material than in the human RNA sample.

We next asked which protocol would allow to detect the widest possible range of different miRNAs. As this is likely to depend on the sequencing depth, we considered the same number of reads (one million) for all libraries sequenced. The numbers of miRNAs mentioned are mean values from at least two independent experiments. With TS1, 690 known human miRNAs were identified, which represents ~ 27% of the total number in miRBase (2588 human miRNAs) (Fig. 3c). The lack of detection of the other miRNAs may be explained either by the fact that the other miRNAs were absent from the starting material or that they escaped detection. While the protocols TS2, − 3 and − 4 detected less miRNAs than TS1, a modest increase in miRNA detection was seen with TS5, and − 6, capturing 714 and 730 miRNAs, respectively. A more marked increase was obtained with the NEXTflex protocols; Nf1, − 2, and − 5 captured 827, 816 and 773 miRNAs, respectively. The SMARTer protocol, strikingly, performed relatively poorly with only 565 miRNAs detected. These results thus indicate that with the standard TruSeq protocol a substantial proportion of the miRNAs present in the starting material for library preparation escape detection at a sequencing depth of 1 million sequences, and that the NEXTflex protocols improve the capture of miRNAs.

**Fig. 4:**
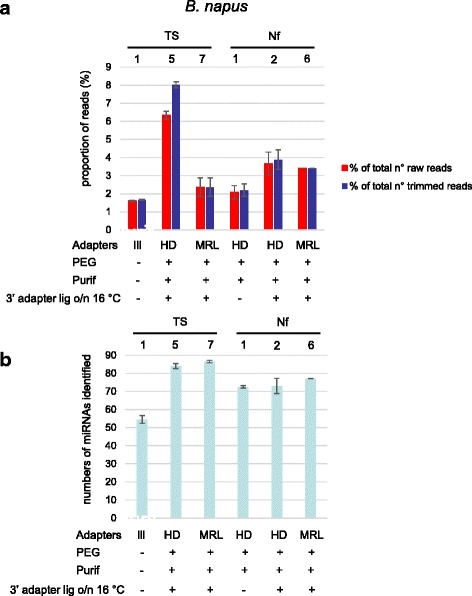
**a** Percentage of reads mapping to *Brassica napus* miRNAs. The proportion of reads mapping to *B. napus* (oilseed rape) miRNAs (with 2’ OMe modification) in miRBase were determined for TruSeq protocols TS1-, 5 and 7, and the NEXTflex protocols (Nf1, 2 and 6). We calculated the percentage of the total numbers of raw reads (red bars) or the total numbers of reads after trimming (blue bars) that mapped to miRNAs. Shown are the mean values of at least two independent experiments and the error bars represent standard deviations. **b** Numbers of *B. napus* miRNAs identified. We determined the numbers of known miRNAs identified with the different protocols. For each protocol, 0.5 million reads were mapped to *B. napus* miRNAs in miRbase (92 in total). Shown are the mean values of at least two independent experiments with standard deviations represented by error bars

For *Arabidopsis*, the TS1 protocol detected 142 known miRNAs, which represents ~ 33% of the total number of miRNAs registered in miRBase (427). As for the human miRNAs, protocols TS2 and TS4 performed less well, but in contrast TS3 performed better than TS1, with 201 miRNAs detected. Another difference was that for *Arabidopsis* the best results were obtained with protocol TS6 (217 miRNAs identified). Both in the TruSeq as well as in the NEXTflex kit, MRL adapter performed better than HD adapters (compare TS5 with TS6, and Nf2 with Nf6). The SMARTer protocol detected 161 miRNAs, slightly better than TS1. To validate these observations, the same experiment was done with *Brassica napus* (oilseed rape) RNA (except that protocols TS2, 3, 4, and S were not included). Very similar results were obtained as for *Arabidopsis*, underscoring the reproducibility of the data (Fig. 4).

It should be noted that the numbers of miRNAs detected are without any threshold of read number per miRNA, and many of the miRNAs detected are covered by only one single read. We also examined the numbers of human or *Arabidopsis* miRNAs detected that have enough coverage to allow accurate detection of expression changes. We determined the numbers of miRNAs detected at thresholds of 10, 20, or 30 reads per miRNA (Additional file 8: Figure S8). The numbers of detected miRNAs significantly decreased for all protocols, but the differences among the protocols did not change.

To further substantiate the observations made with the plant miRNAs, we studied the representation of piRNAs, which are 2’ OMe modified [1], in the human libraries. The reads with 19–24 nt inserts were mapped to the piRNA database (see Methods) and the proportion of mapped reads and the numbers of different piRNAs detected with each protocol were determined (Additional file 9: Figure S9). Similar to the plant miRNAs and in contrast to the human miRNAs, the proportion of mapped reads increased with the “low bias” protocols (but remained low) as compared to TS1, and the numbers of detected piRNAs also slightly increased. These results confirm the notion that the low bias protocols have an improved detection of 2’ OMe RNAs.

In summary, there are substantial differences among the various protocols in sRNA capture. For (unmodified) human miRNAs the standard NEXTflex protocol performed best, whereas modification of the TruSeq protocol led to only a modest improvement. For the (2’ OMe modified) plant miRNAs, modification of the TruSeq protocol led to a more marked improvement of detection and the best results were obtained with MRL adapters in both kits.

### Differences in sRNA capture do not decrease when sequencing at greater depth

**Fig. 5:**
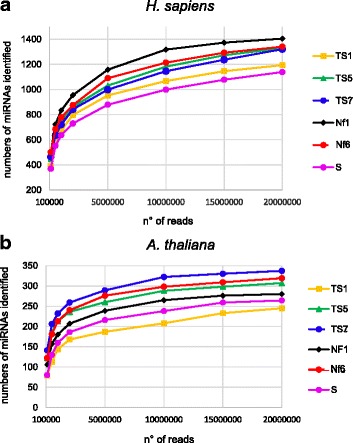
The differences in detection sensitivity among protocols do not change at increased sequencing depth. Sequencing libraries were prepared using three TruSeq protocols (TS1, TS5 and TS7), two NEXTflex protocols (Nf1- and 6), and the SMARTer protocol (S) with human (**a**) or *Arabidopsis* (**b**) sRNA. A total of 20 million sequences were generated for each library. For each protocol, 0.1, 0.5, 1, 2, 5, 10, 15 or 20 million reads were trimmed, the reads with 19–24 nt inserts were mapped to miRBase and we calculated the number of identified miRNAs

We next wished to determine whether the differences in miRNA detection among the protocols would persist at greater sequencing depths or if, when sequencing deeply enough, a similar number of miRNAs would be detected with all protocols. We therefore sequenced a series of human and *Arabidopsis* libraries following protocols TS1, − 5, − 7, Nf1, − 6, and S, and generated 20 million reads for each library. We determined the number of known miRNAs identified at increasing read numbers ranging from 100 thousand to 20 million reads (Fig. 5a and b). For all protocols, the number of identified miRNAs strongly increased along with read numbers up to ~ 2 million reads and then continued to increase more slowly up to 20 million reads. Importantly, the increase in miRNA detection follows a very similar trend for all protocols and the differences in the number of detected miRNAs remain practically the same at each given read number. These observations indicate that even with the best performing protocols coverage of the miRNAs present in the libraries remains incomplete at 20 million sequences; indeed, there are still many miRNAs covered by only one read, confirming the lack of saturation (not shown). Thus, for all protocols, more sequences would be required to reach saturation, but the less well performing protocols would need much deeper sequencing than the better performing protocols. This is illustrated by the fact that, to detect 250 different *Arabidopsis* miRNAs, 20 million reads are required with protocol TS1, whereas with protocol TS7 only 2 million reads are sufficient (Fig. 5b).

We have thus established that some protocols detect much larger numbers of different miRNAs than others at the numbers of sequences generated. However, we previously observed that each protocol has its specific biases (even the protocols with relatively low levels of bias) causing some sRNAs to be strongly detected while others are underrepresented. It is therefore possible that each protocol has its own specific subpopulation of miRNAs that are well represented. To address this question, we compared the miRNA expression profiles for the different protocols. Additional file 10: Table S1 shows the proportion of reads corresponding to all miRNAs detected with the various protocols and a heat map representation of the 50 best detected miRNAs is shown in Additional file 11: Figure S10. Rather different profiles were observed for the various protocols. The profiles cluster as a function of the type of adapter used; TS5, Nf1, and Nf2 (using HD adapters) cluster together as do TS7 and Nf6 (using MRL adapters), consistent with adapter-specific sequence bias. However, TS1 and TS3, although both using standard Illumina adapters, have rather different profiles, indicating that PEG addition in TS3 changes the expression profile.

We asked if a combination of different protocols might help to further increase the number of different miRNAs detected. To test this possibility, we compared the number of different human miRNAs detected with 1 million of reads for protocol Nf1 (827, Fig. 3c), or for a combination of protocols TS1, TS5, TS7, Nf1, and Nf6 (200 K reads each; 1 million in total). With this combination we detected 796 different miRNAs (data not shown). Thus, while combining different protocols may allow the capture of some miRNAs that would escape detection when using each individual protocol alone, the total number of detected miRNAs was not greater than for protocol Nf1.

## Conclusions

In this study, we searched for a sRNA library preparation protocol with the lowest possible levels of sequence- and 2’ OMe bias. The novel SMARTer smRNA-seq kit from Clontech and the CATS kit from Diagenode had less bias than the TruSeq and NEXTflex kits, probably owing to the absence of ligation steps. However, surprisingly, these kits also produced large amounts of side products and as a result did not perform better for the detection of biological miRNAs. The use of MRL adapters and PEG led to bias reduction in both the TruSeq and NEXTflex kit but also increased side product formation. For the detection of human (unmodified) miRNAs the standard NEXTflex protocol performed best, and the modifications we introduced had little effect. In contrast, for plant miRNAs (2’ OMe modified) these modifications substantially improved detection, and the best results were obtained with PEG and MRL adapters in both the TruSeq and the NEXTflex kit. Our results thus yield improved conditions for the detection of 2’ OMe RNAs and in addition indicate that different protocols work best for different types of sRNA.

It should be mentioned here that we have not exhaustively tested all sRNA library preparation kits available on the market. However, at least to our knowledge, none of these other kits use randomised adapters and are therefore likely to have more bias than the NEXTflex, SMARTer and CATS kits. It should be kept in mind that despite the strong bias, the standard TruSeq protocol can quantitatively detect even small expression changes of both normal and 2’ OMe RNAs, provided that these are well detected.

## Methods

### Oligonucleotides

All oligonucleotides used in this study were purchased from Sigma-Aldrich or BIOO Scientific (HPLC purified). A series of six synthetic small RNAs, RNA 1–6, was purchased from Sigma-Aldrich. Of each RNA a 2’-OMe variant (RNA-OMe 1–6) carrying a single nucleotide (nt) substitution (to distinguish the 2’-OMe variant from the unmodified RNA) was purchased. To predict the secondary structures of RNAs1–6 alone, linked to the 3’ Illumina adapter, or linked to both the 3′ and 5’ Illumina adapters (as after 3′ and 5′ adapter ligation, respectively), we used the Mfold web server (http://unafold.rna.albany.edu/?q=mfold) [25]. The structures with the adapters provide an idea of the structures formed in the ligation reaction mixtures as the sRNAs and the adapters are expected to co-fold [7].

A mix of random 21 nt RNA oligonucleotides was purchased from BIOO Scientific. All of these RNAs carried a 5′ monophosphate. RNAs 1–6 and their 2’ OMe variants were added to the mix of random RNAs to a final concentration of 0.1 μM each (1% of the total, concentration of the total mix: 10 μM).

In some of the library preparation protocols (see below), the adapters included in the kits were replaced by custom adapters; we used custom HD adapters and MRL adapters. The 3′ adapters were pre-adenylated following a protocol described by Chen et al. [26]; 1 nmol of 5′ phosphorylated oligonucleotides were incubated with T4 RNA ligase 1 and 1 mM ATP at 20 °C overnight. All custom adapters were purified from 15% denaturing acrylamide gels followed by ethanol precipitation and dissolved in water to a final concentration of 10 μM. After purification, aliquots of the adapters were again migrated on 15% denaturing acrylamide gels for quality control. See Additional file 12: Table S2 for oligonucleotide sequences and modifications.

### RNA extraction

Individual plants for the two plants species *Arabidopsis thaliana* (ecotype Col-0) and *Brassica napus* (oilseed rape, variety Darmor) were grown under controlled conditions (18 °C during 8 h at night and 21 °C during 16 h during the day) in the same climate chamber. Samples were collected all at once for each species. Total RNAs were extracted from young leaves and stems or flower buds using the TRIzol® reagent following the manufacturer’s protocol (Invitrogen), with addition of 0.2 μL/mL beta-mercapto-ethanol to TRIzol® extemporarily. Human RNA (HeLa) was from the Total RNA-seq kit from ThermoFisher.

### Library preparation

All protocols used in this study were based on the Illumina TruSeq Small RNA Sample Preparation kit, the BIOO Scientific NEXTflex Small RNA Sample Preparation kit V2, the New England Biolabs NEBNext kit, the SMARTer smRNA-seq kit from Clontech, or the CATS kit from Diagenode. As starting material either the above-described mix of synthetic RNAs was used (10 pmol RNA per reaction) or biological small RNA samples. Biological small RNA samples were prepared as follows: 10 μg of total RNA from *Arabidopsis*, oilseed rape or human (HeLa) was migrated on 15% polyacrylamide gels containing 8% urea alongside with molecular size markers. RNA was visualized with SYBR Gold (Life Technologies) and 15–30 nt RNAs were excised from the gel. The gel pieces were crushed by centrifugation using 0,5 mL Eppendorf tubes with tiny holes at the bottom. These tubes were put in 1,5 mL Eppendorf tubes followed by 2 min centrifugation at 13 krpm. Subsequently 300 μL 0,3 M NaCl was added to the crushed gels and RNA was eluted by rotation overnight at 4 °C. RNA was ethanol precipitated and resuspended in 15 μL water. We used 1 μL of synthetic RNA mix (10 pmol, or about 70 ng) or 1 μL of gel-purified biological small RNA (~ 0,7 ng) for library preparation.

*TS1. TruSeq – standard conditions*. Library preparation was done using the Illumina TruSeq Small RNA library preparation kit following the manufacturer’s instructions, except that for 3′ adapter ligation 1 μL of synthetic or biological small RNA was mixed with 1 μL RA3 adapter followed by 2 min’ denaturation at 70 °C. After this denaturation step the samples were put on ice and 4 μL of water, 2 μL of ligation buffer, 1 μL of RNA ligase 2 truncated (New England Biolabs) and 1 μL of RNaseOUT was added.

*TS2. TruSeq + HD adapters.* The same procedure as for TS1 except that the Illumina adapters RA3 and RA5 were replaced by HD adapters.

*TS3. TruSeq + PEG*. The same procedure as for TS1 except that after the denaturation step for 3′ adapter ligation 4 μL of PEG8000 was added instead of water and ligation was done at 16 °C overnight instead of 1 h at 28 °C.

*TS4. TruSeq + PEG + HD adapters.* The same procedure as for TS3 except that the Illumina adapters RA3 and RA5 were replaced by HD adapters.

*TS5. TruSeq + PEG + HD adapters + purification.* The same procedure as for TS4 except that, instead of adding STOP solution, a purification step was performed after 3′ adapter ligation to get rid of excess 3′ adapter. Two rounds of purification were done using AMPure beads (Beckman Coulter) as described in the protocol of the BIOO Scientific kit, except that the ligation products were eluted in 9 μL H2O instead of 11 μL. Subsequently 2 μL of Illumina ligation buffer was added for 5′ adapter ligation.

*TS6. TruSeq + MRL adapters.* The same procedure as for protocol TS2 except that MRL adapters were used instead of HD adapters.

*TS7. TruSeq + PEG + MRL adapters + purification.* The same procedure as for protocol TS5 except that MidRand adapters were used instead of HD adapters.

*Nf1. NEXTflex protocol.* Library preparation was done using the BIOO Scientific NEXTflex Small RNA-Seq kit V2 following the manufacturer’s instructions with 3′ adapter ligation for 2 h at 22 °C.

*Nf2. NEXTflex protocol*: as for protocol Nf1 but with 3′ adapter ligation overnight at 16 °C.

*Nf3. NEXTflex protocol w/o HD adapters*: the adapters from the NEXTflex kit were replaced by the Illumina adapters.

*Nf4. NEXTflex protocol w/o PEG*: PEG was replaced by water.

*Nf5. NEXTflex protocol + MRL adapters w/o PEG*: the adapters from the BIOO kit were replaced by MRL adapters and PEG was replaced by water.

*Nf6. NEXTflex protocol + MRL adapters*: the adapters from the BIOO kit were replaced by MRL adapters.

*NN. NEBNext protocol*: Library preparation was done following the manufacturer’s instructions with 3′ adapter ligation for 1 h at 25 °C.

*S. SMARTer protocol*: Library preparation was done using the standard protocol provided with the kit. For the libraries from the synthetic RNA mix ATP was added to the polyadenylation reactions and 7 cycles of PCR were done. For the libraries from the biological samples the polyadenylation reactions were done without supplemented ATP and 13 cycles of PCR were done.

*C. CATS protocol*: Library preparation was done following the manufacturer’s instructions.

### Next-generation sequencing and bioinformatics analyses

All sequencing was done using Illumina platforms, either the MiSeq or the NextSeq500 instrument. For each protocol, several independent libraries were prepared; the mean values are represented in the Figures. Cutadapt [27] version 1.14 was used to remove standard Illumina and MRL adapter sequences. The minimum overlap between the read and the adapter was set to 4 nucleotides, and reads shorter than 10 nucleotides were discarded. Python scripts were used to trim HD adapters and also the random 3-base sequences upstream of the small RNA inserts for the SMARTer protocol. The microRNA databases were downloaded from http://www.mirbase.org/ftp.shtml (download date July 16, 2015); we only used the mature sequences of *Arabidopsis thaliana*, *Homo sapiens* and *Brassica napus*. For piRNA detection, the reads of the human libraries were mapped to piRBase (http://www.regulatoryrna.org/database/piRNA/; downloaded March 2, 2017). The sampling of the reads was done using seqtk, version 1.0-r31 (H. Li, https://github.com/lh3/seqtk/). Only the reads with a length between 19 and 24 nt were kept using python scripts. Mapping to the databases was performed with Bowtie2, version 2.1.0, [23] allowing no mismatches. Python scripts were used to count the number of miRNAs detected.

For the annotation of the short side products (10–18 nt inserts) obtained with the SMARTer libraries from human or *Arabidopsis* RNA, the obtained sequences were trimmed for adapter sequences and were mapped to various databases. For human, the sequences were mapped to the database of small human non-coding RNAs (DASHR; downloaded on October 5, 2017) [28], mirBase (for partial miRNA sequences), and the regulatory RNA database (http://www.regulatoryrna.org/database/piRNA/download/archive/v1.0/fasta/) for piRNA sequences. The *Arabidopsis* sequences were mapped to databases for various non-coding RNAs;ftp://ftp.ensemblgenomes.org/pub/release-24/plants/fasta/arabidopsis_thaliana/ncrna/Arabidopsis_thaliana.TAIR10.24.ncrna.fa.gz), tRNAs (http://gtrnadb2009.ucsc.edu/Athal/Athal-tRNAs.fa) and miRNAs (miRbase). All of these databases were downloaded on October 6, 2017.

To generate the heat map shown in Additional file 11: Figure S10 we used the heatmap.2 function from the R package gplots (R version 3.4.2).

## Additional files

Additional file 1: Figure S1. Histograms representing the percentage of the total numbers of raw reads (before trimming) corresponding to RNA(OMe)1–6 with the TruSeq protocols TS1–7, the NEXTflex protocols Nf1–6, the NEBNext protocol (NN), the SMARTer protocol (S) and the CATS protocol. Blue bars represent the numbers of reads corresponding to each individual RNA, red bars represent the numbers of reads corresponding to RNA1–6 (*total RNA*) or RNA-OMe1–6 (*total RNA-OMe*). Shown are the mean values of at least two independent experiments. Error bars represent standard deviations. Note that in the absence of bias or loss of sequences, for each individual RNA the percentage of the total number of reads should be 1%, and for the sum of the unmodified or the 2’ OMe RNA this percentage should be 6%. For each RNA the ratio of the read numbers for the unmodified- and the 2’ OMe variant is indicated below the histograms (2’ OMe bias; in yellow). (PDF 1.01 mb)

Additional file 2: Figure S2. Box plot representation of the proportion of reads (%) corresponding to RNAs 1–6 with the various protocols. In the ideal situation, 16,7% of the reads (indicated by a red line) should correspond to each RNA, without significant variability among the different RNAs. The data for each individual replica of the various protocols are shown. Horizontal black bars indicate the median RNA (MR). (PDF 1.01 mb)

Additional file 3: Figure S3. Polyacrylamide gel analysis of ligation products of RNA1, RNA1-OMe, RNA4 and RNA6. Samples were taken after 3′ and subsequent 5′ adapter ligation followed by electrophoretic separation on a 10% denaturing polyacrylamide gel. Mixtures of the synthetic RNAs and 3′ adapter without ligation (−) were migrated along with the ligation products. Unligated 3′ adapter and synthetic RNAs, which almost co-migrated in the gels, are indicated by an accolade. Asterisks indicate from bottom to top: unligated 5′ adapter, RNA ligated with 3′ adapter, RNA ligated with 5′ adapter, and RNA ligated with both adapters. Note that RNA ligated with 3′ adapter migrates faster than RNA ligated with 5′ adapter because the 3′ adapter (21 nt) is smaller than the 5′ adapter (26 nt). (PDF 1.01 mb)

Additional file 4: Figure S4. Histograms representing the percentage of the total numbers of raw reads corresponding to RNA(OMe)1–6 with (A) the TruSeq protocol (TS1), (B) the NEXTflex protocol (Nf2), and (C) the SMARTer protocol (S). Green bars represent the results obtained with the standard numbers of PCR cycles (11 cycles for TS1, 14 cycles for Nf2, and 7 cycles for S), blue bars represent 50 additional cycles of PCR, and red bars represent the standard number of PCR cycles but using AMV reverse transcriptase instead of Superscript II for cDNA synthesis. (PDF 1.01 mb)

Additional file 5: Figure S5. Assessment of quantitative detection of synthetic RNAs with protocols TS1, TS5, Nf2, or S. Libraries were prepared from a synthetic RNA mixture in which RNA(OMe)1–6 were each at 1% final concentration, supplemented with random 21 nt RNAs (mix A). Alternatively, an RNA mixture was used in which the concentrations of RNA(OMe)1–6 were changed (mix B); see table 1 for details. The coefficients of determination (R^2^) were determined for the fold changes obtained with each protocol for the unmodified RNAs (R^2^_OH,_ blue dots) and for the 2’OMe RNAs (R^2^_OMe,_ red dots) separately, and for the collection of the unmodified and 2’ OMe RNA together (R^2^_tot_). The fold changes obtained with the various protocols were compared with the theoretical values and the different protocols were compared to each other. (PDF 1.01 mb)

Additional file 6: Figure S6. Quantitative detection of oilseed rape miRNAs using protocols TS7, Nf2, and Nf6. Libraries were preparing from *B. napus* small RNA preparation originating either from floral buds or from stems and leaves and miRNA expression changes were measured. The detected fold changes with the different protocols were compared to each other and the coefficients of determination (R^2^) were calculated. (PDF 1.01 mb)

Additional file 7: Figure S7. Annotation data for the short side products (10–18 nt inserts) with human and *Arabidopsis* libraries. After adapter trimming the human sequences were mapped to the database of small human non-coding RNAs (DASHR), mirBase (for partial miRNA sequences), and the regulatory RNA database for piRNA sequences. The *Arabidopsis* sequences were mapped to databases for various non-coding RNAs (Ensemblgenomes), tRNAs (Genomic tRNA database) and miRNAs. See Methods for details. (PDF 1.01 mb)

Additional file 8: Figure S8. Numbers of known human or *Arabidopsis* miRNAs identified with different thresholds of coverage. We determined the numbers of known miRNAs identified with the various protocols for human or *Arabidopsis* with a minimum coverage of 1 read per miRNA as shown in Fig. 3. Alternatively, we set thresholds at minima of 10 (green bars), 20 (yellow bars), or 30 (pink bars) reads per miRNA. For each protocol, one million of reads were trimmed and the 19–24 nt inserts were used for mapping. Shown are the mean values of at least three (human) or two (*Arabidopsis*) independent experiments with standard deviations represented by error bars. (PDF 1.01 mb)

Additional file 9: Figure S9. Numbers of known human piRNAs identified. We determined the numbers of known piRNAs identified with the various protocols. For each protocol, one million of reads were trimmed and the 19–24 nt inserts were used for mapping to human piRNAs in piRBase (see Methods for details). Shown are the mean values of at least two independent experiments with standard deviations represented by error bars. (PDF 1.01 mb)

Additional file 10: Table S1. (XLSX 154 kb)

Additional file 11: Figure S10. Heat map representation of miRNA expression profiles obtained with protocols TS1, TS3, TS5, TS7, Nf1, Nf2, Nf6, and S. We determined the proportion of reads mapping to each miRNA as a percentage of the total number of mapped reads. These proportions are represented by a colour spectrum from very light red (weak expression) to dark red (strong expression). Shown here are the results for the 50 most highly expressed miRNAs, and the sequences and names of the miRNAs are indicated on the right. Data for all detected miRNAs are shown in Additional file 2: Table S2. (PDF 1.01 mb)

Additional file 12: Table S2. Oligonucleotides used in this study. (XLSX 9 kb)

## Abbreviations

2’-OMe: 2’ O-methyl
HD adapters: High definition adapters
miRNA: microRNA
MRL adapters: MidRand-Like adapters
Nf: NEXTflex
NGS: Next-generation sequencing
PEG: Polyethylene glycol
S: SMARTer
sRNA: Small RNA
TS: TruSeq
