# Supplementary figures and images for: Systematic comparison of small RNA library preparation protocols for next-generation sequencing
Source: BMC Genomics. 2018 Feb 5;19:118. doi: 10.1186/s12864-018-4491-6 (PMC5799908; doi:10.1186/s12864-018-4491-6)

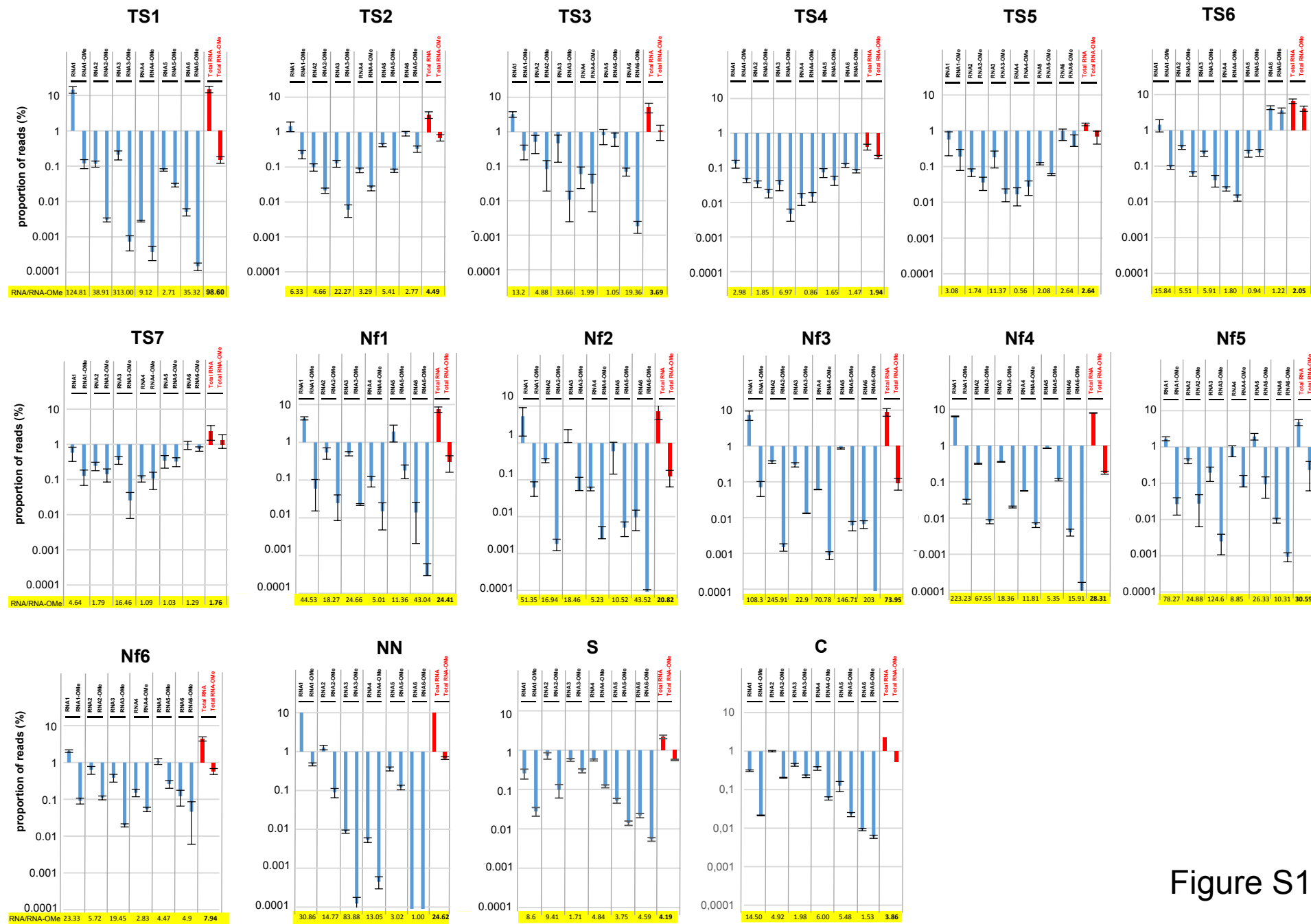

Figure S1

Supplement: Supplementary file 1 — Histograms representing the percentage of the total numbers of raw reads (before trimming) corresponding to RNA(OMe)1–6 with the TruSeq protocols TS1–7, the NEXTflex protocols Nf1–6, the NEBNext protocol (NN), the SMARTer protocol (S) and the CATS protocol. Blue bars represent the numbers of reads corresponding to each individual RNA, red bars represent the numbers of reads corresponding to RNA1–6 (total RNA) or RNA-OMe1–6 (total RNA-OMe). Shown are the mean values of at least two independent experiments. Error bars represent standard deviations. Note that in the absence of bias or loss of sequences, for each individual RNA the percentage of the total number of reads should be 1%, and for the sum of the unmodified or the 2’ OMe RNA this percentage should be 6%. For each RNA the ratio of the read numbers for the unmodified- and the 2’ OMe variant is indicated below the histograms (2’ OMe bias; in yellow). (PDF 1.01 mb) [file 12864_2018_4491_MOESM1_ESM.pdf]

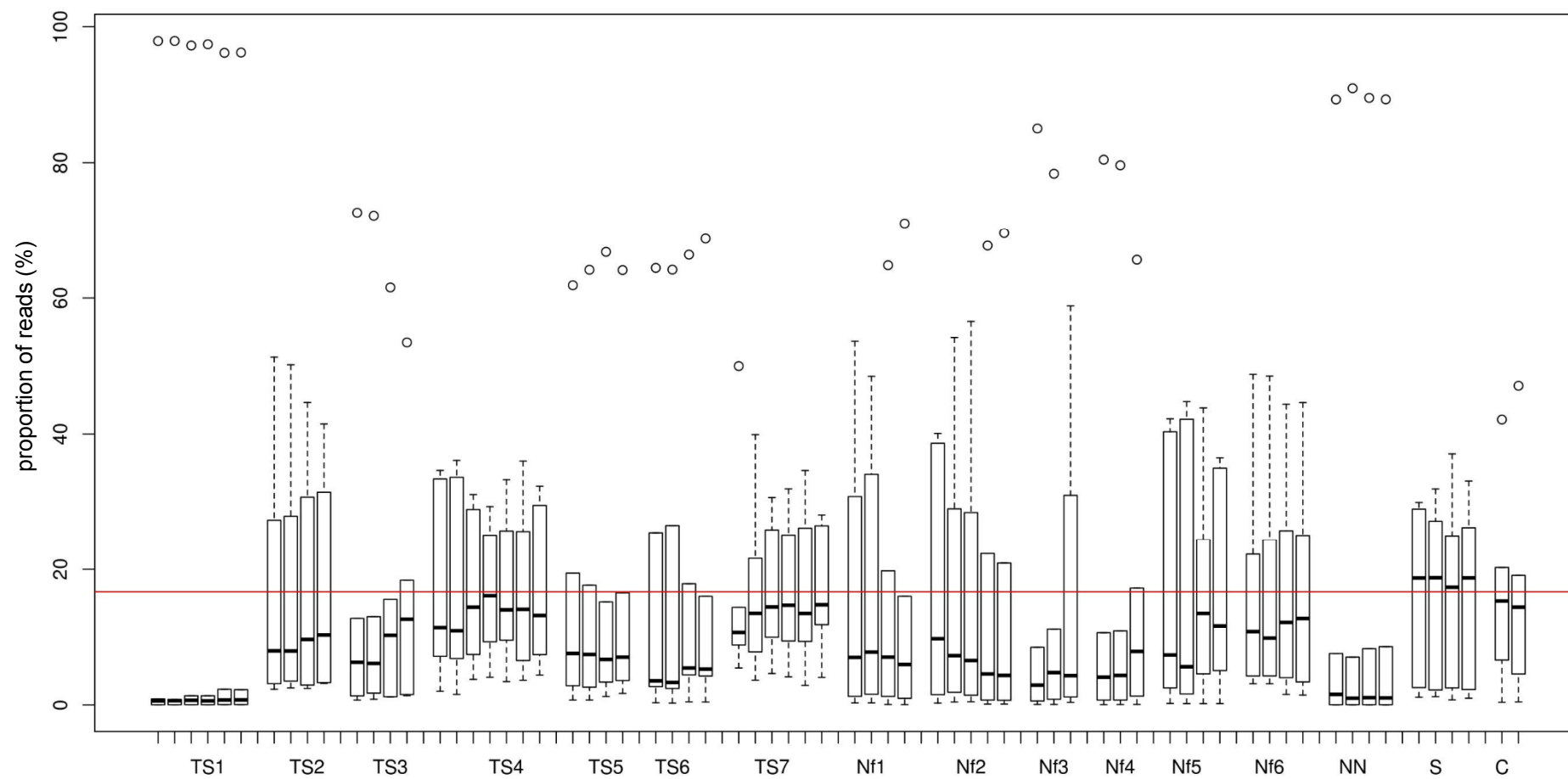

Figure S2

Supplement: Supplementary file 2 — Box plot representation of the proportion of reads (%) corresponding to RNAs 1–6 with the various protocols. In the ideal situation, 16,7% of the reads (indicated by a red line) should correspond to each RNA, without significant variability among the different RNAs. The data for each individual replica of the various protocols are shown. Horizontal black bars indicate the median RNA (MR). (PDF 1.01 mb) [file 12864_2018_4491_MOESM2_ESM.pdf]

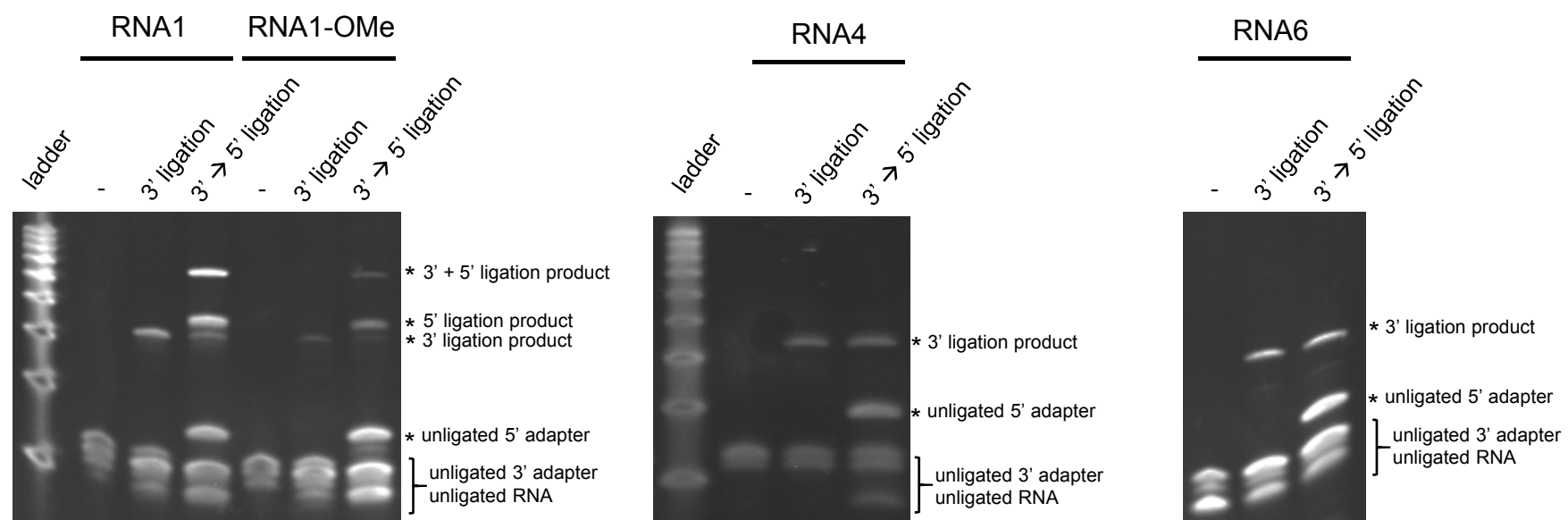

Figure S3

Supplement: Supplementary file 3 — Polyacrylamide gel analysis of ligation products of RNA1, RNA1-OMe, RNA4 and RNA6. Samples were taken after 3′ and subsequent 5′ adapter ligation followed by electrophoretic separation on a 10% denaturing polyacrylamide gel. Mixtures of the synthetic RNAs and 3′ adapter without ligation (−) were migrated along with the ligation products. Unligated 3′ adapter and synthetic RNAs, which almost co-migrated in the gels, are indicated by an accolade. Asterisks indicate from bottom to top: unligated 5′ adapter, RNA ligated with 3′ adapter, RNA ligated with 5′ adapter, and RNA ligated with both adapters. Note that RNA ligated with 3′ adapter migrates faster than RNA ligated with 5′ adapter because the 3′ adapter (21 nt) is smaller than the 5′ adapter (26 nt). (PDF 1.01 mb) [file 12864_2018_4491_MOESM3_ESM.pdf]

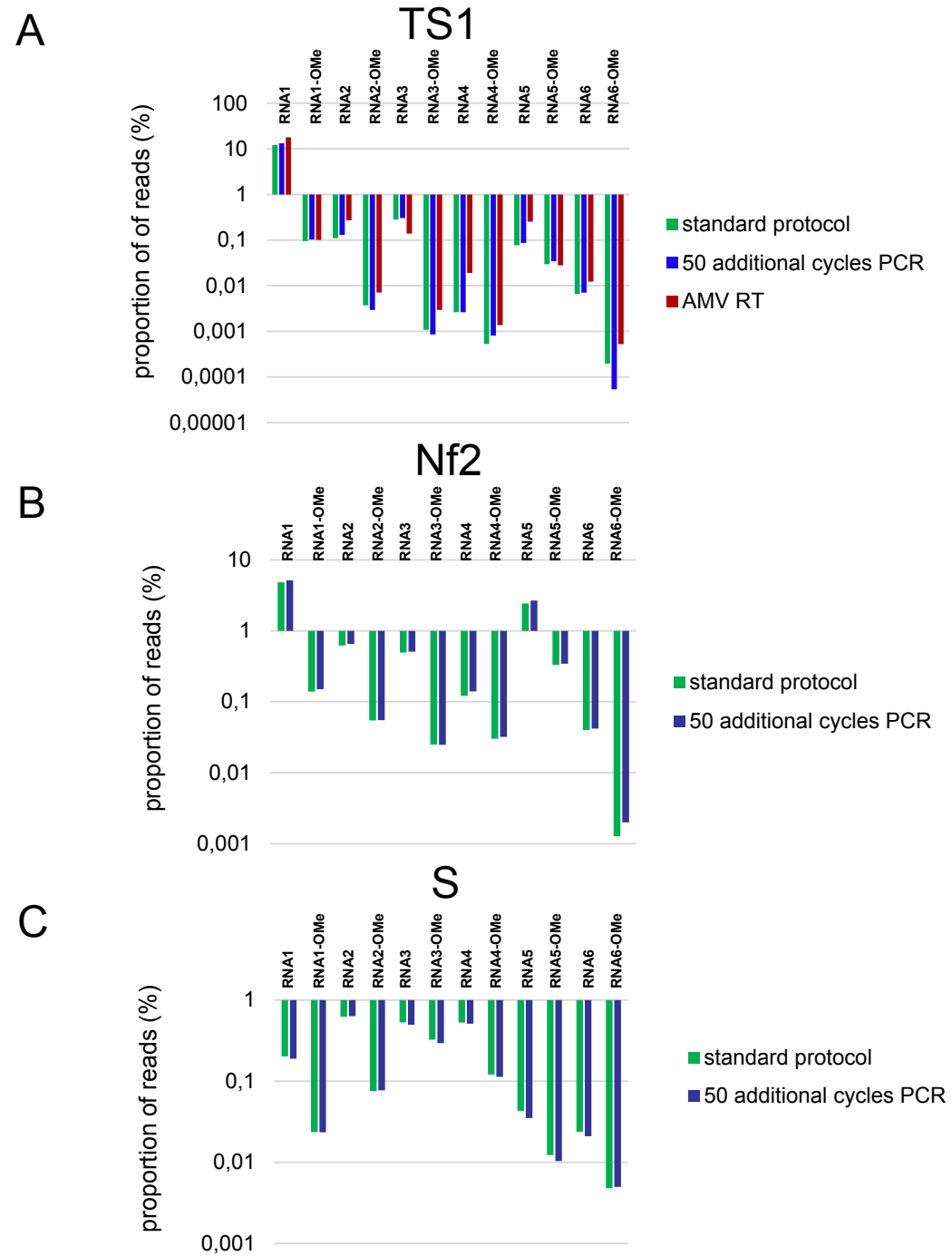

Figure S4

Supplement: Supplementary file 4 — Histograms representing the percentage of the total numbers of raw reads corresponding to RNA(OMe)1–6 with (A) the TruSeq protocol (TS1), (B) the NEXTflex protocol (Nf2), and (C) the SMARTer protocol (S). Green bars represent the results obtained with the standard numbers of PCR cycles (11 cycles for TS1, 14 cycles for Nf2, and 7 cycles for S), blue bars represent 50 additional cycles of PCR, and red bars represent the standard number of PCR cycles but using AMV reverse transcriptase instead of Superscript II for cDNA synthesis. (PDF 1.01 mb) [file 12864_2018_4491_MOESM4_ESM.pdf]

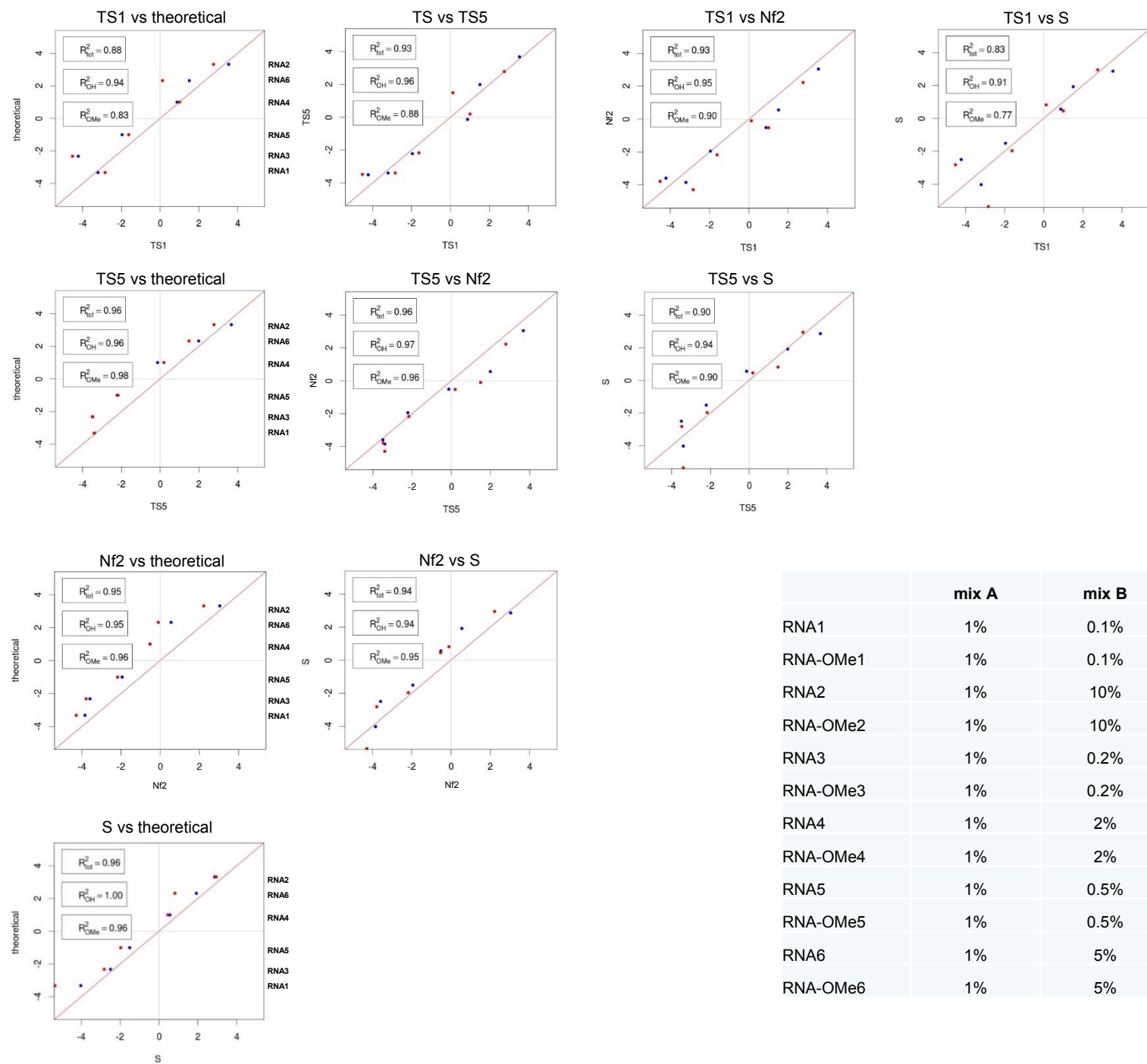

Figure S5

Supplement: Supplementary file 5 — Assessment of quantitative detection of synthetic RNAs with protocols TS1, TS5, Nf2, or S. Libraries were prepared from a synthetic RNA mixture in which RNA(OMe)1–6 were each at 1% final concentration, supplemented with random 21 nt RNAs (mix A). Alternatively, an RNA mixture was used in which the concentrations of RNA(OMe)1–6 were changed (mix B); see table 1 for details. The coefficients of determination (R2) were determined for the fold changes obtained with each protocol for the unmodified RNAs (R2OH, blue dots) and for the 2’OMe RNAs (R2OMe, red dots) separately, and for the collection of the unmodified and 2’ OMe RNA together (R2tot). The fold changes obtained with the various protocols were compared with the theoretical values and the different protocols were compared to each other. (PDF 1.01 mb) [file 12864_2018_4491_MOESM5_ESM.pdf]

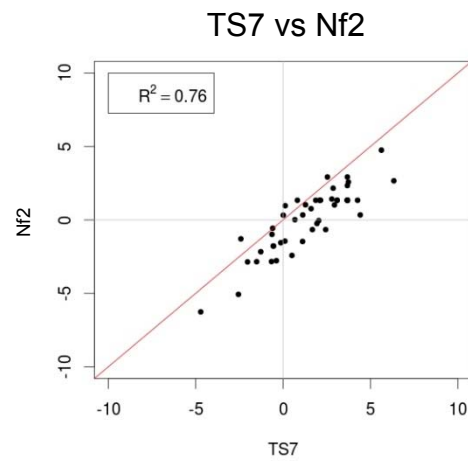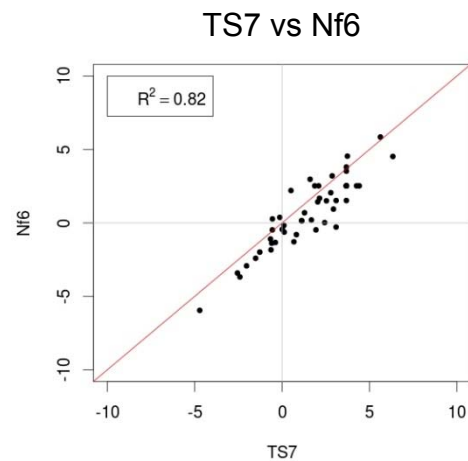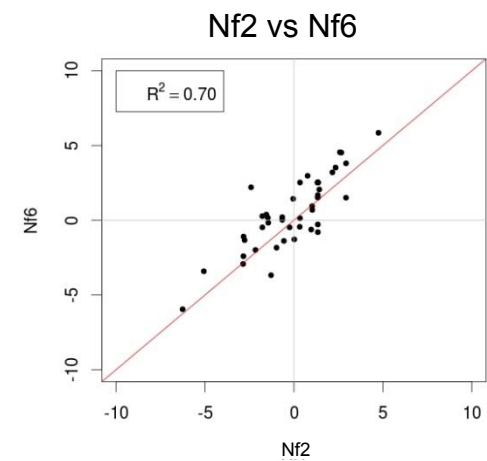

Figure S6

Supplement: Supplementary file 6 — Quantitative detection of oilseed rape miRNAs using protocols TS7, Nf2, and Nf6. Libraries were preparing from B. napus small RNA preparation originating either from floral buds or from stems and leaves and miRNA expression changes were measured. The detected fold changes with the different protocols were compared to each other and the coefficients of determination (R2) were calculated. (PDF 1.01 mb) [file 12864_2018_4491_MOESM6_ESM.pdf]

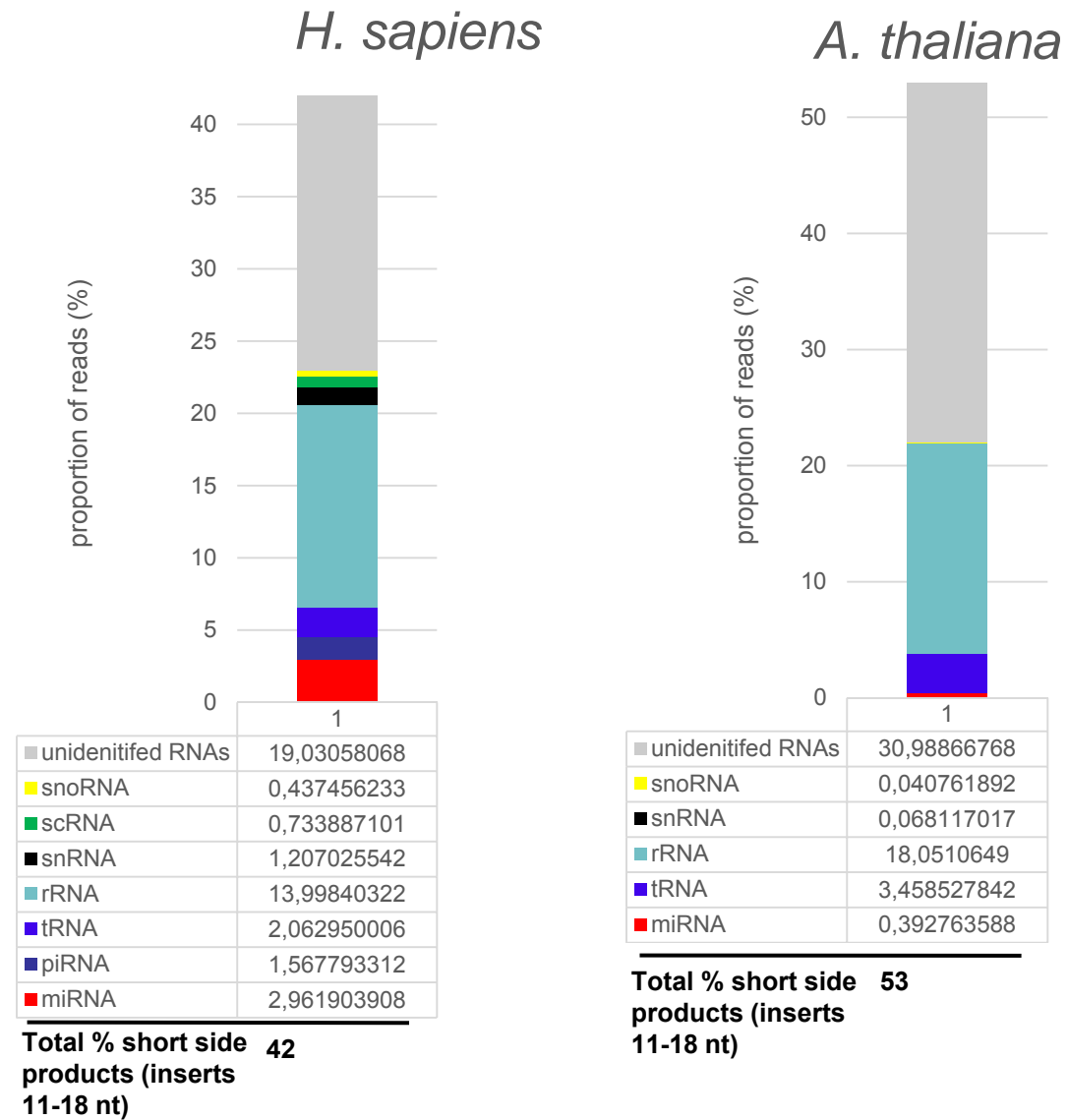

Figure S7

Supplement: Supplementary file 7 — Annotation data for the short side products (10–18 nt inserts) with human and Arabidopsis libraries. After adapter trimming the human sequences were mapped to the database of small human non-coding RNAs (DASHR), mirBase (for partial miRNA sequences), and the regulatory RNA database for piRNA sequences. The Arabidopsis sequences were mapped to databases for various non-coding RNAs (Ensemblgenomes), tRNAs (Genomic tRNA database) and miRNAs. See Methods for details. (PDF 1.01 mb) [file 12864_2018_4491_MOESM7_ESM.pdf]

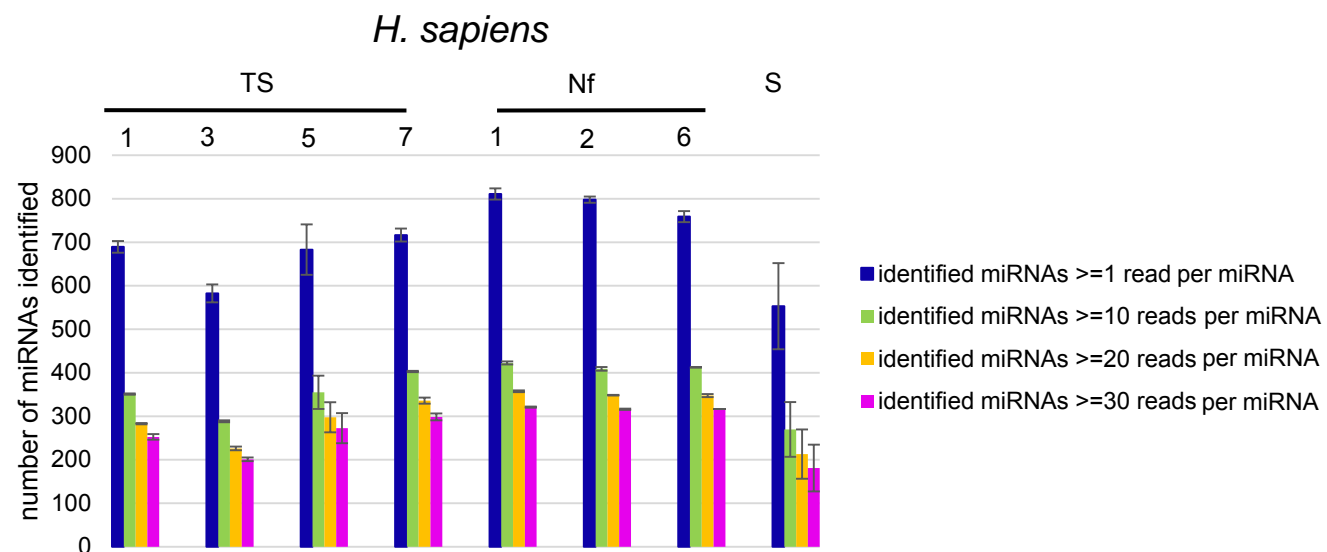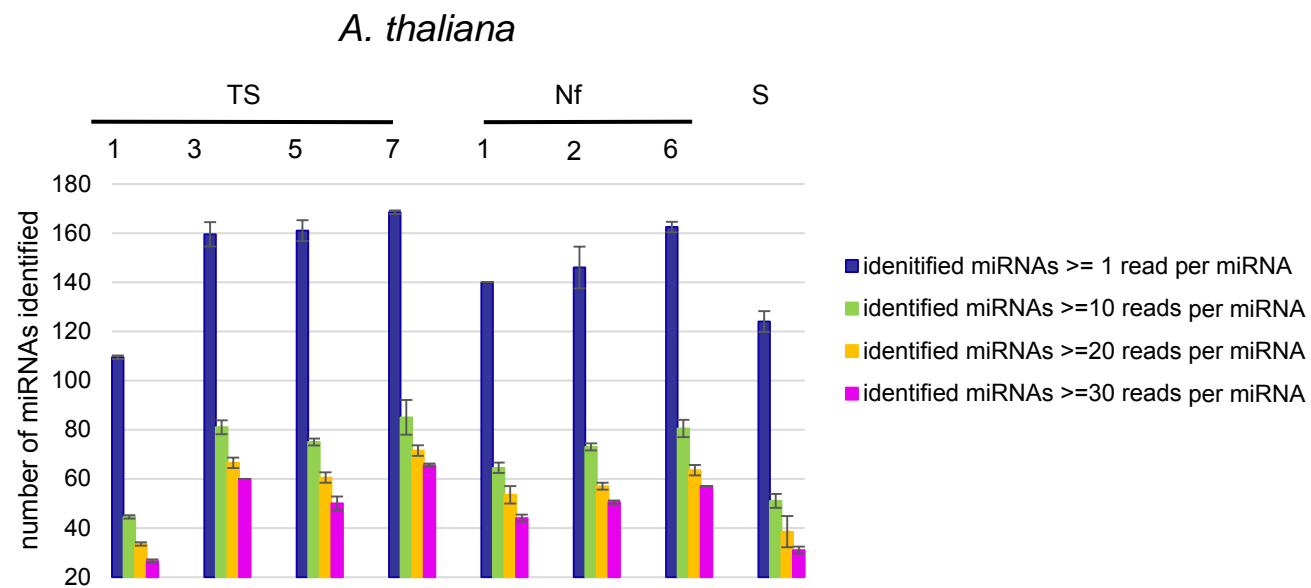

Figure S8

Supplement: Supplementary file 8 — Numbers of known human or Arabidopsis miRNAs identified with different thresholds of coverage. We determined the numbers of known miRNAs identified with the various protocols for human or Arabidopsis with a minimum coverage of 1 read per miRNA as shown in Fig. 3. Alternatively, we set thresholds at minima of 10 (green bars), 20 (yellow bars), or 30 (pink bars) reads per miRNA. For each protocol, one million of reads were trimmed and the 19–24 nt inserts were used for mapping. Shown are the mean values of at least three (human) or two (Arabidopsis) independent experiments with standard deviations represented by error bars. (PDF 1.01 mb) [file 12864_2018_4491_MOESM8_ESM.pdf]

**A**

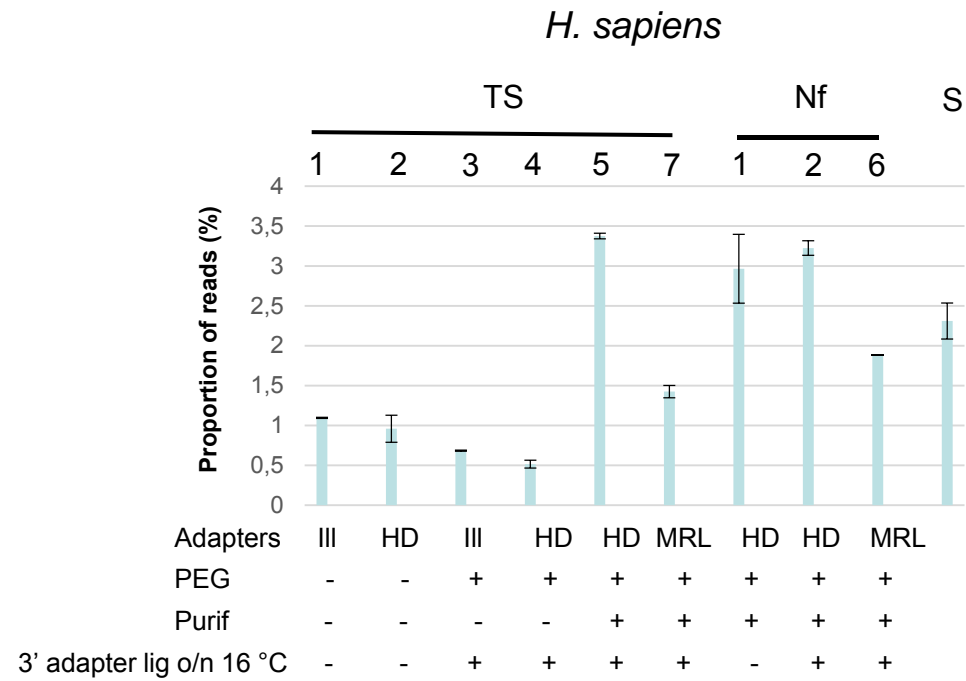

**B**

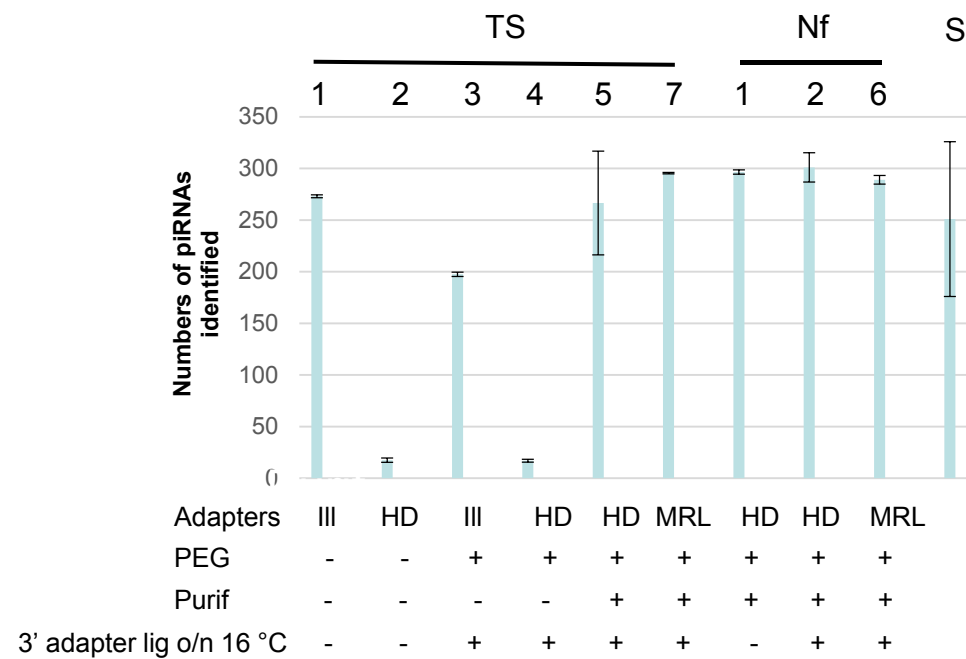

Figure S9

Supplement: Supplementary file 9 — Numbers of known human piRNAs identified. We determined the numbers of known piRNAs identified with the various protocols. For each protocol, one million of reads were trimmed and the 19–24 nt inserts were used for mapping to human piRNAs in piRBase (see Methods for details). Shown are the mean values of at least two independent experiments with standard deviations represented by error bars. (PDF 1.01 mb) [file 12864_2018_4491_MOESM9_ESM.pdf]

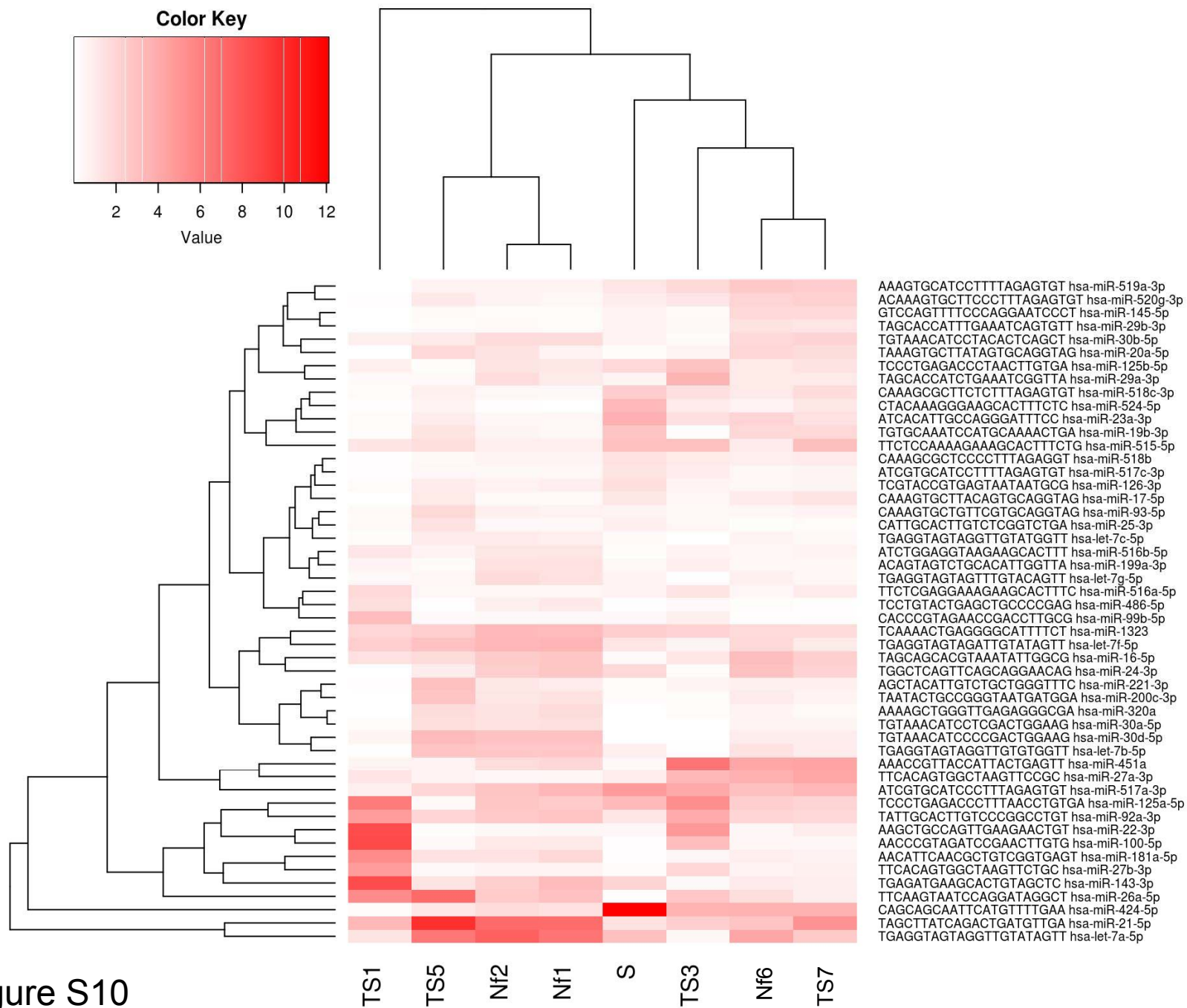

Figure S10

Supplement: Supplementary file 11 — Heat map representation of miRNA expression profiles obtained with protocols TS1, TS3, TS5, TS7, Nf1, Nf2, Nf6, and S. We determined the proportion of reads mapping to each miRNA as a percentage of the total number of mapped reads. These proportions are represented by a colour spectrum from very light red (weak expression) to dark red (strong expression). Shown here are the results for the 50 most highly expressed miRNAs, and the sequences and names of the miRNAs are indicated on the right. Data for all detected miRNAs are shown in Additional file 2: Table S2. (PDF 1.01 mb) [file 12864_2018_4491_MOESM11_ESM.pdf]
